# Trust learning in the repeated trust game: A meta‐analytic study

**DOI:** 10.1111/bjop.70045

**Published:** 2025-12-15

**Authors:** Caitlin Duncan, Lorena Sganzerla, Laura Kaltwasser, Isabel Dziobek

**Affiliations:** ^1^ Berlin School of Mind and Brain Humboldt‐Universität zu Berlin Berlin Germany; ^2^ Department of Psychology Faculty of Life Sciences Humboldt‐Universität zu Berlin Berlin Germany; ^3^ School of Liberal Arts Faculty of the Arts, Social Sciences and Humanities University of Wollongong Wollongong New South Wales Australia

**Keywords:** meta‐analysis, repeated trust game, trust learning

## Abstract

Trust involves making oneself vulnerable by relying on the expectation that others will reciprocate and act in a trustworthy manner, leading to mutual benefit. In behavioural economics and psychology, the Trust Game (TG) is a widely used paradigm to measure trust. The repeated TG is a modified version of the TG in which participants encounter the same partner(s) multiple times, allowing for reputation and trust learning. The aim of the present meta‐analysis was to identify features of the repeated TG, participant characteristics, and manipulations of partner trustworthiness that affect trust learning. This is the first meta‐analytic study to specifically assess trust learning in the repeated TG and included 404 effect sizes from over 8000 participants from 68 studies. Our findings indicate that the partners' behavioural trustworthiness, in the form of their reciprocation rate, is by far the most influential factor in participant trust learning (β = 3.0). Furthermore, the results reveal that manipulating prior information about partners can have an effect on the amount of learning, but only for manipulations of trustworthiness/morality. Notably, in ingroup–outgroup studies, participants learn from their partners' trustworthiness and it is *not* affected by their partners' group membership.

## BACKGROUND

### Trust and the trust game

Drawing from psychology and economics, in behavioural economics, trust can be defined as relying on the expectation that others will reciprocate and act in a trustworthy manner, leading to mutual benefits (Berg et al., 1995). Indeed, trust involves three components: the self, the partner, and the specific goal or expectations in a current situation, that is, ‘I trust you to do X’ (Simpson, 2007). As such, trust can be defined as a willingness to take risks and engage in cooperative behaviour with others in situations characterized by uncertainty and/or risk. Trust therefore involves a certain vulnerability – faith that the recipient of that trust will respond in kind.

Trust can be defined along several axes. First, general trust, or initial trust, is effectively the amount of trust someone demonstrates towards someone they have no information about (McKnight et al., 1998; Yamagishi, 2011; Yamagishi et al., 2015). However, once they have information about the trustworthiness of that individual, their general trust is replaced by evidence‐based trust, tailored to that individual (Yamagishi, 2011; Yamagishi et al., 2015).

The Trust Game (TG) has emerged as one of the most widely used paradigms in the social sciences to study trust and cooperation (Berg et al., 1995) and can be used to measure general and evidence‐based trust. The game involves two players: the trustor and the trustee, or investor and investee, respectively. The trustor is given a sum of money and can choose to send a portion of it to the trustee. The amount sent is multiplied by a factor and the trustee receives the multiplied investment. The trustee can then decide how much, if any, of the multiplied amount to return to the trustor. The amount and/or frequency the trustor/investor invests on a given trial reflects the extent to which the trustor trusts the trustee. The trustee's decision to reciprocate by returning a portion of the multiplied amount reflects their level of trustworthiness. More broadly, reciprocity refers to the negative or positive actions in response to someone's fair or unfair actions (Rabin, 1993). In the TG, a trustee's reciprocity or reciprocation rate, for example, their response to their partners' investment, is referred to as their trustworthiness (Berg et al., 1995). As such, trustee behaviour in the TG can be referred to as (un)trustworthy, (un)fair, or reciprocating low or high.

### Trust learning

In its simplest form, the TG consists of this single interaction, also referred to as the single‐shot TG. However, it can also incorporate multiple rounds with the same partners, allowing for the examination of trust and reciprocity dynamics over time (King‐Casas et al., 2005), also referred to as the repeated TG. The longitudinal aspect of the repeated TG contributes to understanding the evolution of trust (Cox & Deck, 2005), reflecting how trust is established through repeated interactions, depending on the trustworthiness of their partners (Anderhub et al., 2002; Camerer & Weigelt, 1988). Typically what is examined and reported in repeated TG studies is either the participants' initial trust on the first trial (Johnson & Mislin, 2011; Stanley et al., 2011) or participants' average investment/trust behaviour with their respective partners during the game (Cañadas et al., 2015; Telga et al., 2018), which does not reflect how they change their trust behaviour over time. This change in trust behaviour over time is what we refer to as *trust learning*. Although trust learning could be defined in different ways, we defined it as the standardized mean difference between participants' initial trust/investment actions and their last trust/investment actions with the same partners in the repeated TG.

Although many studies have used the repeated TG, currently there exists no meta‐analysis or systematic review on how participants learn from their partners as a function of their trustworthiness, and other contextual factors that may influence this learning process, such as demographics (interindividual), game features, or common manipulations carried out by experimenters.

### Game design and participant characteristics

Many studies have shown that participants learn about their partners' trustworthiness through repeated interaction in a repeated TG paradigm by adjusting their investment behaviour to their partners' behavioural trustworthiness (how much and how often they reciprocate) throughout the course of the game (Cañadas et al., 2015; Delgado et al., 2005; Tortosa et al., 2013; Vermue et al., 2018). This indicates that overall participants do learn to trust their partners (and also learn to trust them less if they are untrustworthy).

However, previous findings point to a variety of factors that could affect trust learning. In both the single‐shot (Johnson & Mislin, 2011) and in the repeated TG (Vermue et al., 2018), it has been shown that participants from different countries invest differently. In terms of the TG design, there is mixed evidence that the investor's endowment and the amount by which the investor's investment is multiplied influence participants' investment behaviour (Alós‐Ferrer & Farolfi, 2019; Johnson & Mislin, 2011; Lenton & Mosley, 2011). Some studies include controlled reciprocation rates with fictitious partners, for example, pre‐programmed responses (Phan et al., 2010), whereas other studies let two participants interact ‘live’ with a real person (Cochard et al., 2004). In ‘live’ interactions, participants often adapt a tit‐for‐tat investment strategy, focused on a trial‐by‐trial response to the *behaviour* of their interaction partner (Engle‐Warnick & Slonim, 2004), which may differ from when participants play with pre‐programmed responses.

As such, a main aim of this meta‐analysis was to examine if these factors (and others, which are detailed in the methods section) found to be significant in influencing participants' trust behaviour in single‐shot games, would also be relevant in participants' trust learning in the repeated TG. We hypothesized that participants' trust learning would be primarily influenced by their partners' trustworthiness (as measured by the reciprocation rate), but that demographics and game features could still have an influence and require testing.

### Priors and trust learning

Studies have also shown that prior biases can impair or affect trust learning (Fareri et al., 2015; Fouragnan et al., 2013; Tortosa et al., 2013; Vermue et al., 2018). Studies that had participants play with fair/good (high reciprocation) partners and unfair/bad (low reciprocation) partners have shown a stronger result in reducing participants' prior biases (Chang et al., 2010; Delgado et al., 2005; Telga et al., 2018), compared with those that had participants play with partners who reciprocate randomly (Fareri et al., 2012, 2015). Most studies using the repeated TG show that participants change their trust/investment behaviour to adapt to their partners' trustworthiness, but that pre‐existing priors or biases can still play a role (Chang et al., 2010; Fareri et al., 2012, 2015; Telga et al., 2018; Vermue et al., 2018).

In terms of the cognitive process of trust learning, studies which used reinforcement learning models to assess how participants update their trust towards their partners in the repeated TG differ in their results (Chang et al., 2010; Fareri et al., 2012, 2015). Reinforcement learning is the process or situation where an agent (a person or an algorithm) interacts with an environment, learns from its experiences, and adapts its behaviour to achieve its goals by maximizing the accumulated rewards (Sutton & Barto, 2018). The learning process of that agent can be modelled, including estimating a learning rate. As such, reinforcement learning models are one approach with which participants' learning in the repeated TG can be assessed: participants experience gains and losses in a sequence of moves/bids for trust with the ostensible objective of maximizing gains (Chang et al., 2010).

Related to reinforcement learning is Bayes' Theorem of conditional probabilities, in which a prior or existing belief is integrated with new evidence to create the posterior (updated) belief (Achtziger et al., 2014). The frameworks are complementary: both are ways to model how an agent or person learns in the face of evidence (Achtziger et al., 2014; Sutton & Barto, 2018). Both shed light on the cognitive processes behind learning in the repeated TG, for example, the participant's prior investment can be taken as their prior belief, their partners' reciprocation or defection as evidence, and their subsequent investment represents the updated belief (Chang et al., 2010).

Reinforcement learning models applied to the repeated TG have shown mixed results. Chang et al. (2010) found that both the initial trustworthiness judgement as well as subsequent experience interact synergistically in participants' decisions to trust throughout the game. However, other studies have shown evidence for confirmation and positivity biases (Fareri et al., 2012, 2015), fitting with findings from forced‐choice tasks^1^ outside of the TG literature (Biele et al., 2009, 2011; Lefebvre et al., 2017; Palminteri et al., 2017), indicating that more broadly participants may learn differently from positive outcomes versus negative outcomes. This points to the importance of the partners' reciprocation in trust learning.

Given this mixed evidence about the role of priors, our second area of investigation analysed how negative and positive priors affect trust learning in commonly used manipulations.

### TG manipulations

The following manipulations are used often: the partners' morality or pre‐existing trustworthiness, social closeness/similarity of the partner to the participants, and social group (ingroup–outgroup) membership.

Several studies have included a manipulation of partners' morality or pre‐existing trustworthiness prior to the TG (Delgado et al., 2005; Fareri et al., 2012; Maurer et al., 2018; Zarolia et al., 2017). Such manipulations have been shown to significantly impair participants' ability to learn their partners' true (behavioural) trustworthiness (Zarolia et al., 2017). However, the extent to which the priors affect participants' investment behaviour seems to depend on the strength of the partners' reciprocation or behavioural trustworthiness (Delgado et al., 2005).

Group membership can similarly affect participants' trust learning: one study showed that participants learn less from outgroup members of a different nationality compared with ingroup members of the same nationality with high reciprocity but learn equally from outgroup and ingroup members with low reciprocity (Vermue et al., 2018). Another study (Duncan et al., 2023) has shown that participants changed their trustworthiness judgements according to their partners' behaviour but that the greatest change in investment and in trustworthiness perception was for ethnic ingroup members who behaved unfairly (with low reciprocity). Social closeness may bias participants' learning even more: in one social closeness TG design, participants had a strong bias to trust their friend more than a computer or other social control, even when all were pre‐programmed to have the same reciprocation rate (Fareri et al., 2015).

Given that the literature has shown differing results in terms of how priors interact with partner trustworthiness to influence trust learning, we conducted subgroup analyses on studies that used the following manipulations: social closeness, group membership, prior trustworthiness/morality, and an exploratory analysis of priors across all manipulations.

## METHODS

### Preregistration, collected data and supporting information

The preregistration, summary of collected data (publications and corresponding effect sizes), the code for analysis, and Supporting Information, can all be found on OSF.

### Systematic search

As the TG paradigm is used in psychology, neuroscience, and behavioural economics, we searched the following databases: *Scopus*, *PubMed*, *PsycInfo*, *PsycArticles*, and *EconBiz*. Search terms were applied to ‘all fields’ in each database, including title, abstract, and keywords to be as inclusive as possible.

**FIGURE 1:**
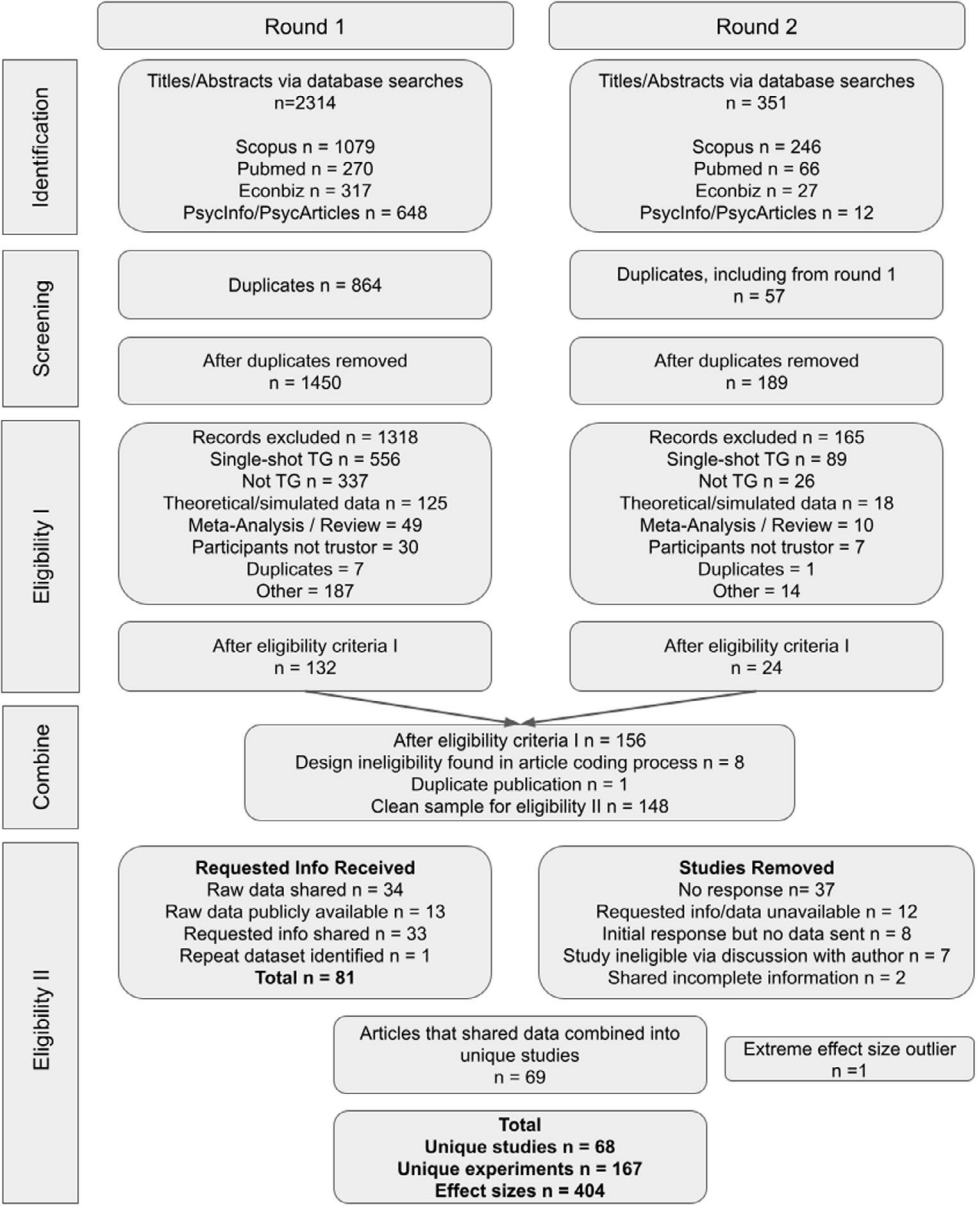
Diagram of study collection, evaluation, and removal.

The search terms were pre‐registered and as follows: “trust game” OR “multi‐round trust game” OR “repeated trust game” OR “investment game” OR “multi‐round investment game” OR “repeated investment game” OR “multi‐round reciprocity” OR “repeated reciprocity”. The following filters were also included: article language – English, article type – journal article or conference paper, and year of publication starting in 1988, the first year the Trust Game was published (Camerer & Weigelt, 1988; later formalized as the investment game by Berg et al., 1995). The search term “reciprocity” was always accompanied by “multi‐round” or “repeated” because a preliminary, investigative search without “multi‐round” or “repeated” yielded thousands of additional, irrelevant results. Figure 1 shows the number of ineligible studies that were removed.

The search was completed on July 14th, 2020. Due to delays in data coding and collection, a second round was conducted on Nov 28th, 2021 to add more contemporary articles to the meta‐analysis. Both searches were identical, except for a minimum publication year of 2020 in the second round to avoid duplicates.

After excluding duplicates and meta‐analyses (Figure 1), the searches yielded 1639 unique articles as potentially eligible for analysis.

#### Inclusion criteria

Following the systematic searches, papers were then evaluated by two independent reviewers (pre‐registered as three reviewers, but one had to leave the project partway) in two phases: Phase I assessed minimal criteria for inclusion based on the study's design; Phase II identified missing data and requested it from the authors.

Phase I – minimal criteria for inclusion

1. Must be the repeated TG, meaning repeated interactions with the same partner(s).
2. Studies published in 1988 or later.
3. Participants must have played the role of the *investor*. If participants played both roles, the data in which they were in the investor/trustor role was analysed
4. Partners are represented as human or human‐like.
5. The repeated interaction needs to be measured on the same day.

From the list of 1639 articles, 148 met the meta‐analysis inclusion criteria. The inter‐rater reliability – a raw proportion of the sum of ratings in agreement divided by the total number of ratings – was 90%. Any reviewer discrepancies related to article eligibility were thoroughly debated before a final decision on inclusion or exclusion was made.

In Phase II of our eligibility assessment, we requested the necessary information from authors to calculate a trust learning effect size (Hedges' *g*, standardized mean difference). For nearly all articles, information needed to be requested or analysed if already available on an open‐source platform. If the information for participants' first and last trial investments or participants' pre‐ and post‐TG trustworthiness ratings of partners, or the Pearson's correlation^2^ between those values was neither reported nor made available to us when requested (nor was the raw data shared), then the study was excluded. Authors were also asked for missing demographic data, such as the proportion of female participants or mean age; however if not provided, we did not exclude the article. Authors were sent one reminder after 4–6 weeks and a second reminder 3 weeks thereafter.

Of the 148 articles meeting inclusion criteria I, effect sizes were obtained for 83% or 56% of those articles. Data published in multiple publications were identified and counted as one study (Figure 1). Regarding data availability, 13 studies had the raw data publicly available; for 24 studies the authors shared their raw data, and for 30 studies, the authors shared the metrics we requested (see Figure 1).

Our final sample included 68 studies or 167 experiments^3^ with 164 unique participant groups,^4^ 404 effect sizes, and a total *N* = 8285 unique participants.

### Coding effect sizes

#### Calculating effect sizes: Hedge's *g*

As pre‐registered, we used a repeated measures design to assess participants' learning, using either average investment on the first trial and the last trial per participant group or pre‐ and post‐TG trustworthiness ratings of their partners, to calculate a standardized mean difference. We used this to be able to accommodate studies that reported participants' pre‐ and post‐TG trustworthiness ratings of their partners. However, few studies did this, so we used first and last trial TG investments. Specifically, Cohen's *d* for repeated measures (Cohen's *d*_*rm*_) was used, as it removes additional bias from the effect size by taking into account the correlated nature of the data for repeated measures (Lakens, 2013) as follows:

$$\text{Cohen}\prime s\;d_{\mathrm{rm}}=\frac{M_{\text{diff}}}{{\mathrm{SD}}_{\mathrm{pre}}^{2}+{\mathrm{SD}}_{\text{post}}^{2}-2\times r\times {\mathrm{SD}}_{\mathrm{pre}}\times {\mathrm{SD}}_{\text{post}}}\times \sqrt{2\left(1-r\right)}$$

where *M*_diff_ is the difference in means, SD is the standard deviation and *r* is the Pearson correlation coefficient between the measurements within the same group of participants at two time points, pre and post. However, when sample averages are used to estimate the population effect, it results in an overestimation of the population effect (Hedges, 1981). To address this bias, Hedges' *g* is used, as it is the equivalent of Cohen's *d* but with bias correction (Cumming, 2013; Hedges & Olkin, 1985; Lakens, 2013), as follows:

$$g\approx d_{\mathrm{rm}}\times \left(1-\frac{3}{4\left(N\right)-9}\right)$$

#### Coding effect sizes and missing data

Correlations between first and last trial investments or pre‐and post‐TG trustworthiness ratings (referred to as pre‐ and post‐measures in the above equations, respectively) were not reported, a value necessary to calculate an unbiased Hedges' *g* effect size (Lakens, 2013). In the preregistration we stated we would impute missing correlation values with the average correlation value obtained from other studies. However, as the standard deviation of correlation values was high and impacted effect sizes substantially, as aforementioned in the Phase II eligibility criteria, we excluded studies for which we could not calculate an unbiased effect size, for example, if neither the necessary values nor the raw data were shared with us.

### TG design features and participant characteristics

The TG design features and participant characteristics that were included in our main models and their justification are described in detail below. More details are in the Code Book and dataset on the OSF repository.

#### Reciprocation rate – pre‐registered

The reciprocation rate quantifies the amount or frequency of the partner's reciprocation. The reciprocation rate has been defined differently across studies. Some authors define it as the proportion reciprocated of the multiplied investment (Buskens et al., 2016), others define it as the proportion reciprocated of the original investment (Acar‐Burkay et al., 2014), and yet others define it as a frequency of a binary reciprocation (Fareri et al., 2015; Phan et al., 2010). To account for the multiplication factor (see below), we defined and analysed the reciprocation rate as the average proportion of the multiplied investment that was reciprocated.

#### Reciprocation type – pre‐registered

Three categories of reciprocation rates were identified: ‘Fixed’ refers to the reciprocation being fixed across the experiment, for example, the partner reciprocates 50% of the investment on 75% of the trials (van den Bos et al., 2012). ‘Variable’ denotes that the reciprocation rate changes, which would include the ‘live’ games (human‐to‐human interaction) with other participants as the reciprocation is by definition not fixed (Johnsen & Kvaløy, 2016) as well as an algorithm‐based reciprocation study that has a defined variability, for example, reciprocating 45%–75% of the multiplied investment or 0%–25% of the multiplied investment (Vermue et al., 2018). ‘Adaptive’ is when the reciprocation rate increases the more the participant invests, or decreases the less the participant invests (Gromann et al., 2013), potentially facilitating the learning process.

#### Counterfactual reciprocation rate – exploratory

One way to facilitate reputation learning is to provide information about the partners' reciprocating behaviour on trials for which the participant does not make an investment, henceforth referred to as counterfactual reciprocation. Palminteri et al. (2017) showed that people take into account factual and counterfactual learning similarly. In a repeated TG that included counterfactual outcomes, Phan et al. (2010) showed that activation in brain areas crucial for reward processing was similar for avoiding a loss (learning from counterfactual information) and receiving reciprocation. Given these results, we hypothesized that including counterfactual outcomes in the repeated TG could facilitate participants' learning.

#### Investment: binary or continuous – pre‐registered

In some studies, participants have a binary option to invest or not invest with a fixed amount (Fareri et al., 2012, 2015; Phan et al., 2010). In others, participants can select any amount from their endowment (Rosenberger et al., 2019, 2020; Vermue et al., 2018), which could affect participants' investment behaviour and learning given that they have a wider range of options to indicate their trust.

#### Multiplication factor – exploratory

The trustor's investment is multiplied by a factor such that the partner receives more than what the participant invested (therefore creating an expectation of reciprocation). Johnson and Mislin (2011) found that it did not play a role in trust (investment from the trustor), but *did* play a role in trustworthiness (reciprocation from the trustee), whereas Lenton and Mosley (2011) found that increasing the multiplier increases the proportion of the endowment sent by the trustor. A higher multiplication factor could lead to higher investment and more learning.

#### Partner endowment – pre‐registered

Johnson and Mislin (2011) found that if the trustee received an initial endowment, trustors invested less with them, whereas Rodrigo‐González et al. (2021) found that trustors invested more with trustees who have higher endowments. Conflict between different motives underlying decision‐making in the TG includes expectations of reciprocity (fairness) and inequality aversion (Brülhart & Usunier, 2012; Rodriguez‐Lara, 2018). Given the mixed evidence and motivations for participants displaying an endowment effect, it was included as a moderator.

#### Initial endowment: fixed, variable, first trial only – exploratory

Given that endowment has previously been shown to affect investment (Johnson & Mislin, 2011), the type of endowment that the participant has – only available on the first trial, fixed or varying across trials – could also have an effect and was included as a moderator.

#### Live game versus algorithm – pre‐registered

We define ‘live’ games as those involving human‐to‐human interaction, as opposed to human‐to‐algorithm interaction. In live games, often used in economics studies, both players are free to decide how much to invest and reciprocate on each trial or adopt a tit‐for‐tat approach, mirroring the behaviour of their partner. Subsequently, the learning curves are different than when users play with an algorithm (often used in psychology studies) with a fixed strategy and endgame effects are often present (Cochard et al., 2004; Johnsen & Kvaløy, 2016; Kanagaretnam et al., 2010; van Miltenburg et al., 2012), suggesting more learning with algorithms.

#### Finite versus infinite game – pre‐registered

There is a distinction between studies which inform participants about the number of trials beforehand (Fareri et al., 2012, 2015) and those that do not (Buskens et al., 2016; Fett et al., 2012), henceforth referred to as finite and infinite paradigms, respectively. It has been shown that when participants do know the number of rounds (‘definite’), they tend to invest less on the last trials because reputation building is over (Engle‐Warnick & Slonim, 2004).

#### Trust game reward – exploratory

Johnson and Mislin (2011) showed that if the subjects were randomly selected for payment instead of guaranteed to be paid based on their TG performance, it had a statistically significant, negative effect on the subjects' investment in the single‐shot TG. We therefore included as a binary factor if participants received a real monetary reward for their behaviour/performance in the repeated TG or if they did not receive a reward for their TG behaviour to test if a similar effect to the one found by Johnson and Mislin (2011) would be present for trust learning.

#### Number of trials – pre‐registered

None of the studies in our meta‐analysis explicitly tested the effect of the number of trials on learning. However, in nearly all of the studies, a general learning curve can be observed: when participants play with consistent partners (e.g., fair or unfair) their investment changes accordingly; in the early trials they quickly invest more/more frequently, reaching a peak, then tapering off and the inverse is observed for unfair partners (Chang et al., 2010; Collins & Juvina, 2021; Fouragnan et al., 2013; Phan et al., 2010; Telga et al., 2018; Vermue et al., 2018). Therefore, we hypothesized that the number of trials would facilitate learning.

#### Participant student population – pre‐registered

Early findings suggested students invest less than non‐students (Bellemare & Kröger, 2003; Fehr & List, 2004); however, no statistically significant meta‐analytic effect in the single‐shot TG was found (Johnson & Mislin, 2011). However, these studies used the single‐shot TG and results may differ in a repeated TG.

#### Participant clinical populations – pre‐registered

There is evidence that non‐neurotypical participants with psychological and neurological diagnoses, invested differently, on average, than non‐clinical controls, suggesting that the learning rate between clinical and non‐clinical groups could be different (Gromann et al., 2013; Maurer et al., 2018; Sutherland et al., 2020) for schizophrenia (Gromann et al., 2013) and autism (Maurer et al., 2018).

#### Country/region – pre‐registered as exploratory

There is evidence that people from some cultures are more trusting than others in the TG (Johnson & Mislin, 2011; Kiyonari et al., 2006). It has also been shown that preferences for risk‐taking, positive and negative reciprocity, and trust, vary by country within the collectivist/independent categories (Falk et al., 2018). Therefore, we grouped the countries by continent as was done by Johnson and Mislin (2011). Sample sizes for each country were quite small and can produce unreliable estimates, so country‐level effects were excluded.

#### Participant gender – pre‐registered as exploratory

It has been shown that there are gender differences in trust behaviour. Falk and Hermle (2018) found that women show a higher preference for trust and a lower preference for risk than men do. In the repeated TG, it was found that men showed more basic trust than women and that when playing with fair and unfair partners, males decreased their trust more over time than females did (Lemmers‐Jansen et al., 2017). Together, the results of these studies indicate that gender could be a moderating effect in trust learning in the repeated TG.

#### Participant age – pre‐registered as exploratory

Several studies have shown that trust increases with age (Fett, Shergill, et al., 2014; Poulin & Haase, 2015; Sutter & Kocher, 2007; van den Bos et al., 2012), and that willingness to trust increases linearly with age in a single‐shot TG paradigm (Sutter & Kocher, 2007). Compared with teenagers, older adults invested more with a trustworthy partner, and showed a stronger decline in trust (less investment) with an unfair partner (Fett, Shergill, et al., 2014). However, Lemmers‐Jansen et al. (2017) did not find any behavioural differences based on participant age (ranging from 16 to 27). This effect may not be consistent across each TG setup.

### Analytical strategy

Mixed‐effects models were used for all meta‐analytic estimates, which are recommended when studies are drawn from different populations (Quintana, 2015) and work better than fixed effects only models with highly nested meta‐analytic data (Fernández‐Castilla et al., 2020). Specifically, different conditions were treated as nested within each experiment, and each experiment was nested in the overall study/publication. This was done following the protocol by Harrer et al. (2021), and using the Metafor package in R (Viechtbauer, 2010).

#### Main analyses

The main analytical model included the aforementioned pre‐registered and exploratory features of the TG and demographics that were identified as potentially affecting trust behaviour.

#### Subgroup analyses

Subgroup analyses included in the preregistration are Group Membership, Social Closeness, Trustworthiness/Morality, and having a clinical diagnosis. These subgroup analyses provide insight on specific *types* of manipulations with negative/positive priors and their interaction with the reciprocation rate, as described in the introduction. To extend these findings, we conducted an exploratory analysis that examined the role of a prior – negative, positive or neutral/none – and its interaction with reciprocation rate across all studies.

## RESULTS

Descriptive summaries of the studies, participant characteristics, and TG features are in Table S1, as well as a forest plot of the pooled effect sizes from each study (Figure S1).

### Full features model

The full features model included the reciprocation rate and all moderators listed in the analytical strategy. The model showed a positive, statistically significant effect of the reciprocation rate, with the largest effect size of β = 3.0, confirming the hypothesis that the reciprocation rate has the largest effect on learning. The only other statistically significant effect was a negative effect for live (human–human) versus programmed games (participants play with an algorithm), for example, that participants in live games invest *less* over time compared with programmed games. This is likely due to endgame effects present in human–human games: participants tend to invest less in the last few rounds and especially in the last round to maximize their gains (Camerer & Weigelt, 1988; Engle‐Warnick & Slonim, 2004). Note that the variables multiplication factor and students (compared with non‐students) approach, but do not reach, statistical significance (*p* = .061 and *p* = .066, respectively) (Table 1).

**TABLE 1:** Full features meta‐analytic model.

Effect	Estimate	SE	95% CI		*p*
			LL	UL	
Intercept	−1.535	0.474	−2.467	−0.604	.001
RR	2.954	0.200	2.560	3.348	.000
RR adaptive	−0.152	0.304	−0.749	0.445	.616
RR variable	0.201	0.240	−0.271	0.673	.403
RR counterfactual	−0.054	0.217	−0.480	0.372	.803
Investment Continuous vs. binary	−0.032	0.207	−0.438	0.375	.878
Multiplication factor	0.246	0.131	−0.011	0.504	.061
Endowment first trial only	−0.873	0.714	−2.277	0.531	.222
Endowment variable	−0.302	0.550	−1.385	0.780	.583
Partner endowment	−0.210	0.228	−0.659	0.240	.359
Live game	−0.615	0.307	−1.219	−0.012	.046
Infinite game	−0.468	0.353	−1.163	0.227	.186
Trust game reward	−0.260	0.214	−0.681	0.161	.225
*N* trials	−0.001	0.003	−0.006	0.004	.572
Continent: Africa	0.096	0.661	−1.204	1.397	.884
Continent: Asia	0.313	0.313	−0.303	0.929	.319
Continent: Australia	−0.031	0.260	−0.544	0.481	.904
Continent: Europe	−0.162	0.217	−0.588	0.264	.456
Continent: South America	1.223	0.759	−0.270	2.716	.108
Mean age	−0.007	0.005	−0.018	0.003	.187
Proportion female participants	0.064	0.229	−0.386	0.514	.780
Students	0.382	0.207	−0.026	0.790	.066
Clinical population	0.117	0.148	−0.175	0.408	.432

Random effects	Estimate	*N* levels
Publications (level 1)	0.197	58
Experiment/Participant group (level 2)	0.000	143
Effect size condition (level 3)	0.129	359

*Note*: AIC = 721.1, BIC = 820.4. There was significant moderation of the moderator effects, *F*(df1 = 22, df2 = 336) = 10.9, *p* < .001 and a significant effect of heterogeneity, QE(df = 336) = 668.8, *p* < .001.

Abbreviations: CI, confidence interval; LL, lower limit; RR, reciprocation rate; precise definitions of all variables are described under TG Design Features and Participant Characteristics; UL, upper limit.

It is important to note that 45 effect sizes were dropped from the analysis due to having missing values for the participant mean age and proportion of participants who were female. The values from 11 studies were dropped: Acar‐Burkay et al. (2014), Attanasi et al. (2019), Cochard et al. (2004), Fiedler and Haruvy (2017), Johnsen and Kvaløy (2016), Kanagaretnam et al. (2010, 2012, 2014), Lamba et al. (2020), Lunawat et al. (2021), Meidinger and Terracol (2012), Schniter and Sheremeta (2014), Schniter et al. (2013); Tomlinson (2012). Note that the Kanagaretnam publications used the same data so it is counted as 1 dataset. Schniter and Sheremeta (2014) and Schniter et al. (2013) used the same data and are counted as 1 dataset.

#### Full features model publication bias

**FIGURE 2:**
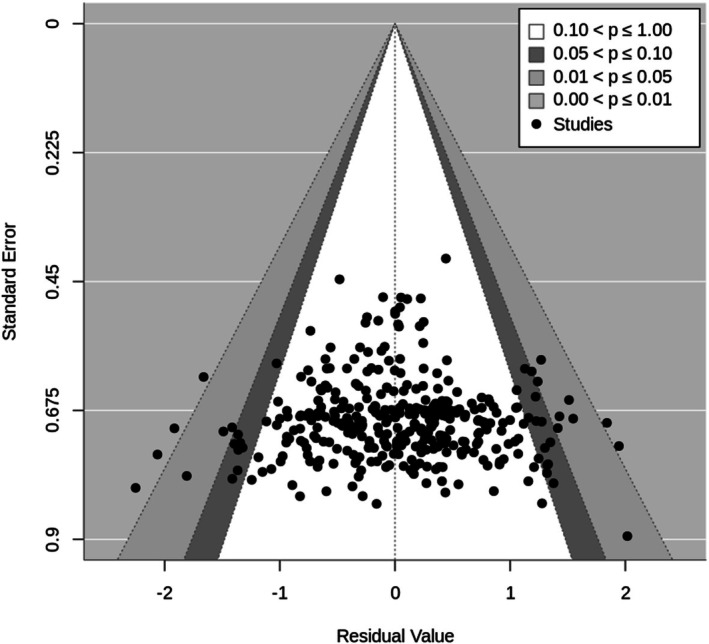
Funnel plot of the full features meta‐analytic model. Each circle represents a learning effect size collected in our study. The learning effect size (Residual Value) is plotted with its associated standard error.

Publication bias was assessed with funnel plots in which asymmetry indicates bias. The funnel plot (Figure 2) shows the learning effect sizes versus the standard error of those effect sizes for each condition within each experiment/study. Ideally, one would observe that the plot is symmetric, particularly in the lower section, where there is high standard error. The plot is symmetric, but there are values that fall outside of the ideal range (white section). The plot shows that studies with larger standard error (less precise) had larger effect sizes than the pooled effect size.

In addition to the funnel plots, an Egger's regression test for publication bias (Egger et al., 1997) was applied by modifying the multilevel mixed‐effects models to include the standard error of the effect sizes as a moderator. If the intercept of the resulting regression significantly deviates from zero, the dataset is considered biased because the relationship between the precision (standard error) and size of studies is asymmetrical (Sterne & Egger, 2005).

The Egger's regression test yielded a statistically significant effect of the intercept, β = −1.4, 95% CI [−2.40, −0.38], SE = 0.51, *t*(335) = −2.7, *p* = .008, indicating possible publication bias. The funnel plot, however, is symmetrical, indicating that at least the publications are not skewed. See Table S2 for all parameters.

### Subgroup analyses

Several subgroup analyses were conducted and the linear mixed models for their meta‐analytic results are summarized in Table 2.

**TABLE 2:** Meta‐analytic results for each subgroup analysis.

Effect	Estimate	SE	95% CI		*p*
			LL	UL	
Group membership model^a^					
Fixed effects					
Intercept	−0.801	0.200	−1.200	−0.402	.000
RR	2.618	0.600	1.422	3.814	.000
Outgroup	−0.241	0.285	−0.809	0.327	.400
RR × Outgroup	0.854	0.859	−0.860	2.567	.324
Random effects					
Level 1: Study (*N* = 8)	0.000				
Level 2: Experiment (*N* = 21)	0.000				
Level 3: Unique effect size (*N* = 74)	0.162				
Social closeness model^b^					
Fixed effects					
Intercept	−0.533	0.464	−1.497	0.432	.264
Distant vs. close partner	−0.189	0.575	−1.385	1.008	.746
Neutral vs. close partner	−0.326	0.574	−1.520	0.867	.576
RR	0.527	1.615	−2.832	3.886	.747
Distant vs. close partner × RR	1.345	1.891	−2.588	5.278	.485
Neutral vs. close partner × RR	1.455	1.888	−2.472	5.381	.450
Random effects					
Level 1: Study (*N* = 3)	0.030				
Level 2: Experiment (*N* = 9)	0.000				
Level 3: Unique effect size (*N* = 27)	0.000				
Trustworthiness/morality model^c^					
Fixed effects					
Intercept	−1.131	0.276	−1.680	−0.583	.000
RR	3.337	0.748	1.853	4.821	.000
Negative vs. neutral partner	0.787	0.334	0.123	1.451	.021
Positive vs. neutral partner	−0.308	0.344	−0.991	0.374	.372
RR × Negative	−2.181	0.912	−3.993	−0.369	.019
RR × Positive	0.010	0.914	−1.805	1.826	.991
Random effects					
Level 1: Study (*N* = 13)	0.051				
Level 2: Experiment (*N* = 32)	0.000				
Level 3: Unique effect size (*N* = 100)	0.455				
Priors and reciprocation rate all studies model^d^					
Fixed effects					
Intercept	−1.153	0.121	−1.391	−0.915	.000
Negative vs. neutral/none prior	0.277	0.171	−0.060	0.613	.107
Positive vs. neutral/none prior	−0.198	0.177	−0.545	0.149	.260
RR	2.723	0.251	2.228	3.217	.000
Negative vs. neutral/none prior × RR	−0.279	0.440	−1.145	0.586	.526
Positive vs. neutral/none prior × RR	0.453	0.436	−0.405	1.311	.300
Random effects					
Level 1: Study (*N* = 68)	0.244				
Level 2: Experiment (*N* = 167)	0.000				
Level 3: Unique effect size (*N* = 404)	0.126				

*Note*: All were mixed‐effects models. By‐study, by‐participant group, and by‐effect size (unique effect size for each condition within a participant group, within a study) random effects were included.

Abbreviations: CI, confidence interval; LL, lower limit; RR, reciprocation rate; UL, upper limit.

^a^ The model fit was AIC = 147.5, BIC = 163.3. There was significant moderation of the moderator effects, *F*(df1 = 3, df2 = 70) = 16.9663, *p* < .0001 and a significant effect of heterogeneity, QE(df = 70) = 122.0476, *p* = .0001.

^b^ The model fit was AIC = 30.5, BIC = 40.0. There was no statistically significant moderation of the moderator effects, *F*(df1 = 5, df2 = 21) = 1.78, *p* = .160 and no significant effect of heterogeneity, QE(df = 21) = 9.519, *p* = .985.

^c^ The model fit was AIC = 210.5, BIC = 233.4. There was a statistically significant moderation of the moderator effects, *F*(df1 = 5, df2 = 94) = 11.617, *p* < .0001 and a statistically significant effect of heterogeneity, QE(df = 94) = 218.3, *p* < .0001.

^d^ The model fit was AIC = 841.2, BIC = 877. There was significant moderation of the moderator effects, *F*(df1 = 5, df2 = 398) = 43.335, *p* < .0001 and a significant effect of heterogeneity, QE(df = 398) = 858.482, *p* < .0001.

#### Group membership – pre‐registered

**FIGURE 3:**
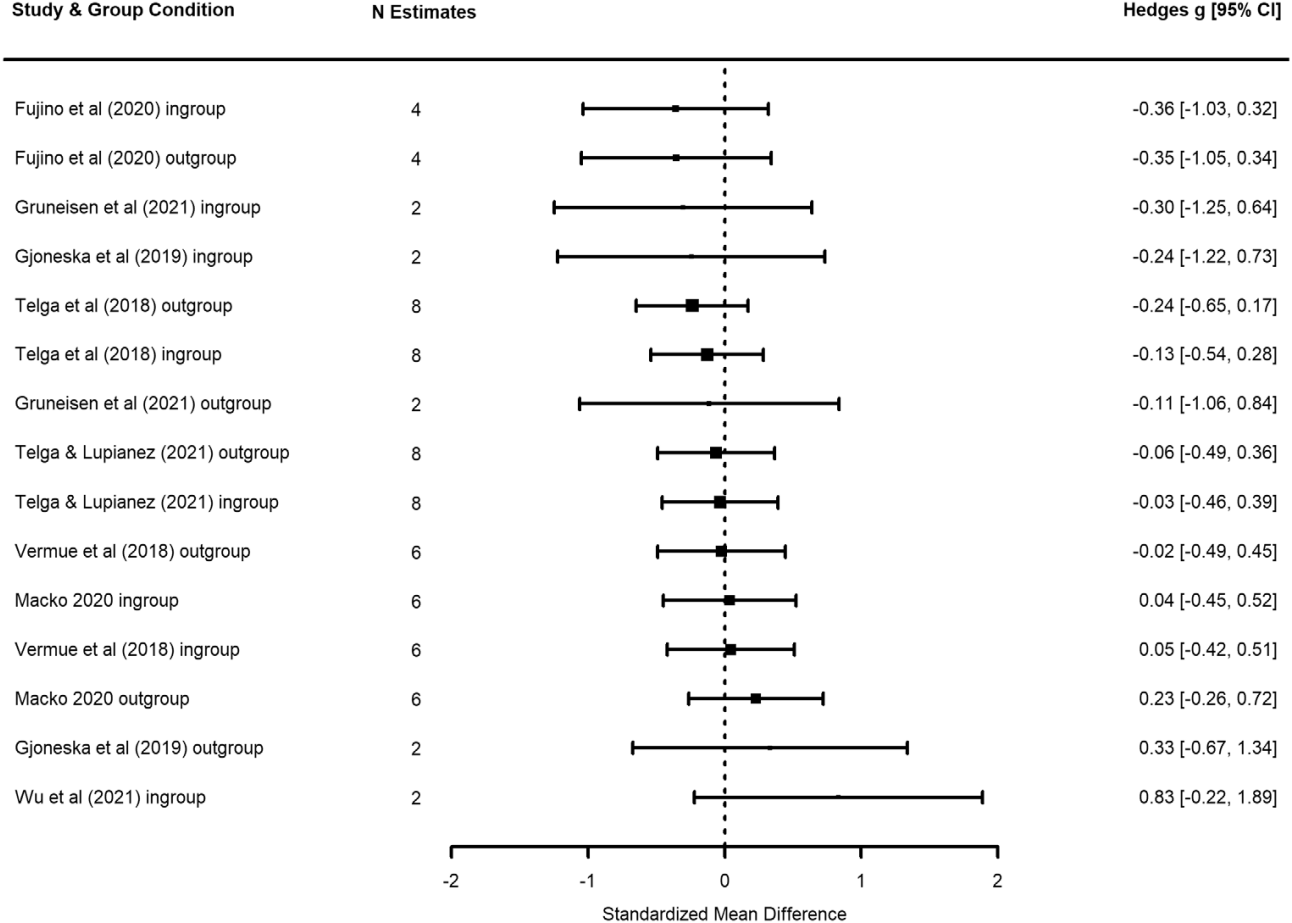
Forest plot of the effect sizes by publication and partner group membership for the group membership analysis. Plotted are the pooled Hedges' *g* and 95% CI for each study, participant group, and condition for the group membership model. *N* estimates = number of effect sizes for a given study and group membership condition. An effect around 0 indicates very little change in trust behaviour or learning occurred. A negative Hedges' *g* indicates participants invested less over time (learning to trust less); a positive Hedges' *g* indicates participants invested more over time (learning to trust more).

This analysis included 74 effect sizes from 21 experiments from eight studies that included an ingroup–outgroup manipulation (Fujino et al., 2020; Gjoneska et al., 2019; Grueneisen et al., 2021; Macko, 2020; Telga et al., 2018; Telga & Lupiáñez, 2021; Vermue et al., 2018; Wu et al., 2021). Each study defined ingroups and outgroups differently; for example, Telga et al. (2018) used ethnicity/race, Vermue et al. (2018) used nationality, Gjoneska et al. (2019) used political beliefs. In all cases, the manipulation is meant to examine if participants interact with ingroup versus outgroup members differently. The meta‐analytic results (Table 2) show that there was no interaction effect of group membership and reciprocation rate, but a statistically significant positive effect of the reciprocation rate. This reflects the conflicting findings of how participants invest with their ingroup and outgroup partners. Figure 3 shows how the effect sizes vary widely for ingroup‐outgroup conditions across studies.

#### Social closeness – pre‐registered

**FIGURE 4:**
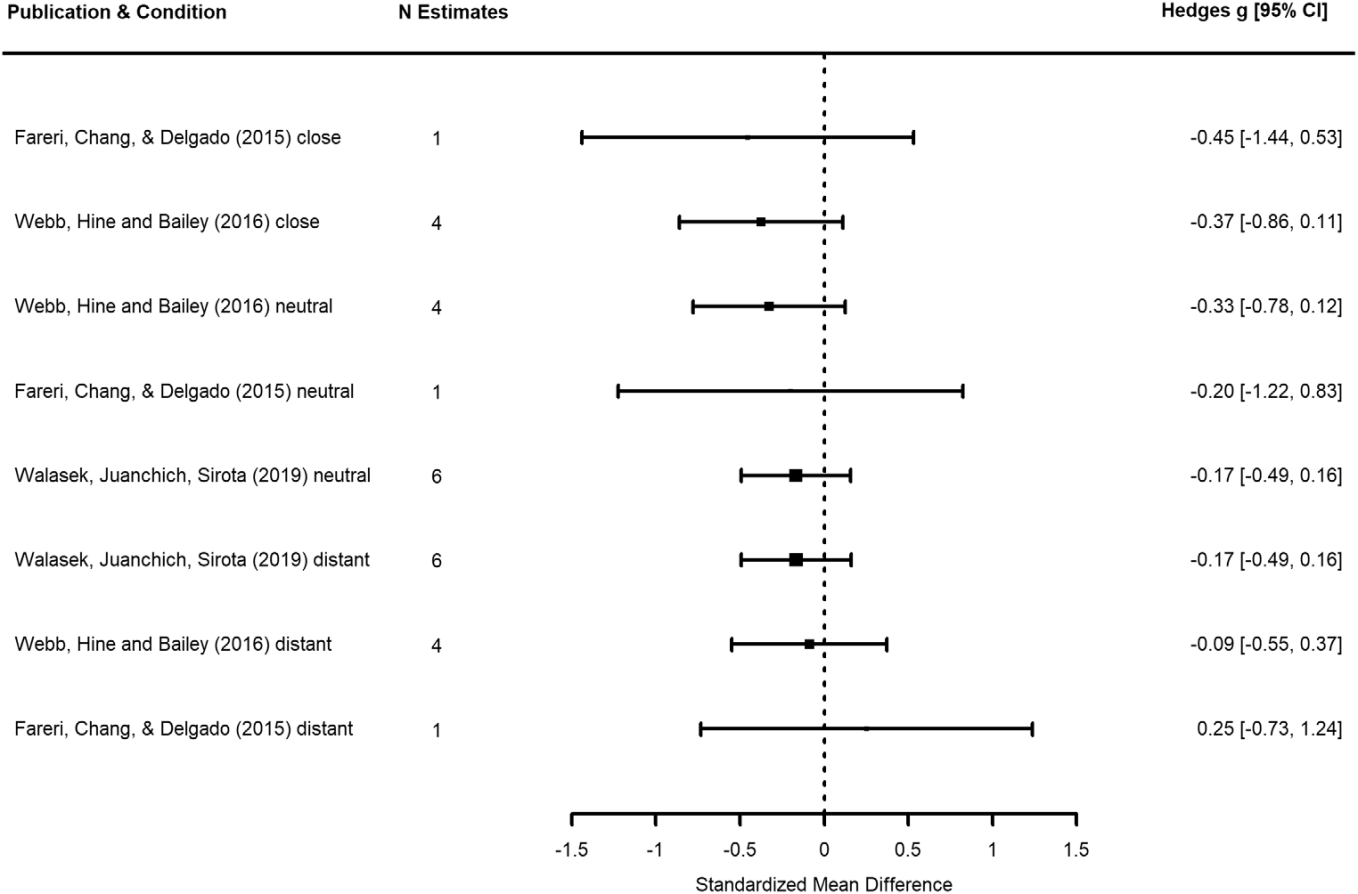
Forest plot of effect sizes by publication and social closeness categories for the social closeness model. Plotted are the pooled Hedges' *g* and 95% CI for each study, participant group, and condition for the social closeness model. *N* estimates = number of effect sizes for a given study and social closeness condition: close, distant, or neutral. An effect around 0 indicates very little change in trust behaviour or learning occurred. A negative Hedges' *g* indicates participants invested less over time (learning to trust less); a positive Hedges' *g* indicates participants invested more over time (learning to trust more).

This subgroup analysis included 15 effect sizes from nine experiments from three studies that specifically manipulated social closeness not part of an ingroup‐outgroup manipulation (Fareri et al., 2015; Walasek et al., 2019; Webb et al., 2016). Three closeness categories were created: close, distant, and neutral, and tested for an interaction effect with the reciprocation rate. The results of the meta‐analytic model (Table 2) reveal no statistically significant effect, including the reciprocation rate. Figure 4 shows the pooled effect sizes for each social closeness category which show similar effects, explaining and illustrating the lack of a social closeness main effect. The groups for this subgroup analysis were smaller than expected, with just 11 effect sizes for distant and neutral categories, and only five for the ‘close’ category. Therefore, this subgroup analysis is likely underpowered to detect effects, especially interaction effects.

To adjust for the small number of samples for the social closeness categories, we ran the model again without the interaction term. Removing the interaction term resulted in an improved model fit (Table S3). Without the interaction term, the model showed a positive, statistically significant main effect of the reciprocation rate, β = 1.67, SE = 0.634, 95% CI = [0.36, 2.98], *p* = .015, and no other statistically significant effects. Figure S2 shows the linear models with and without an interaction term.

#### Trustworthiness/morality

As pre‐registered, we analysed the effects of manipulating partners' trustworthiness or morality apart from their trustworthiness behaviour. This includes manipulations where the partners' prior trustworthiness was manipulated either through communicating that to the participant *or* the participant interacted with the partner and they behaved in a trustworthy/untrustworthy way. It also includes manipulations of morality, for example, giving participants a vignette about the partners' moral behaviour. This resulted in 100 effect sizes, 28 neutral/none, 36 negative, and 36 positive from 32 experiments from 13 studies (Blue et al., 2018; Campellone & Kring, 2013; Fareri et al., 2012; Hooper et al., 2019; Knight et al., 2021; Lamba et al., 2020; Lee et al., 2016; Maurer et al., 2018; Radell et al., 2016; Rățală et al., 2019; Sutherland et al., 2020; Taylor & Stevenson, 2018; Yu et al., 2014).

**FIGURE 5:**
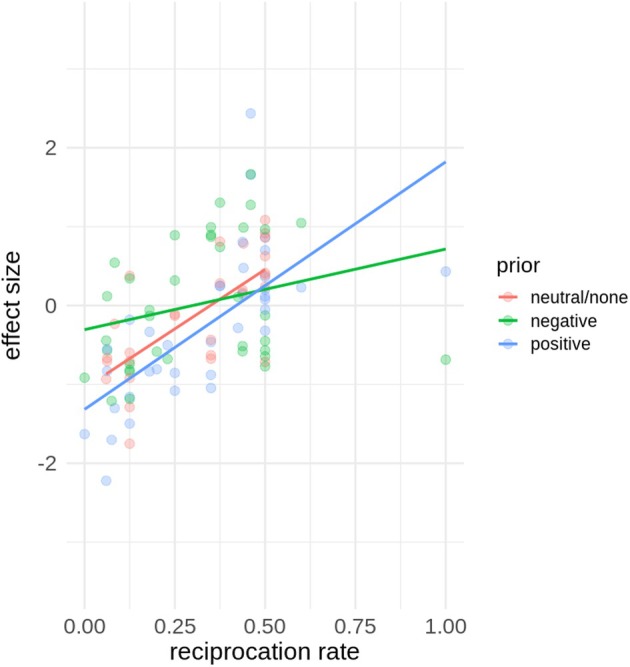
Trustworthiness/morality meta‐analytic model interaction effect. The linear relationship between the reciprocation rate and the corresponding effect size (learning rate) for each type of prior. The circles represent actual data points.

The meta‐analytic model results (Table 2) show a negative interaction effect of negative prior versus none with the reciprocation rate. This indicates that the reciprocation rate has a weaker effect on the learning rate when there is a negative prior compared with when there is a neutral prior/no prior or positive prior (see Figure 5). The model also showed a statistically significant positive main effect of a negative prior and a statistically significant positive effect of the reciprocation rate.

The interaction effect is modelled in Figure 5 which shows that the relationship between the reciprocation rate and effect size changes depending on the prior. For all priors, the effect size increases as the reciprocation increases; however, the slope is different for a negative prior compared with the other priors. At low and high reciprocation rates, there is a larger learning effect (investing less, investing more, respectively) for neutral and positive priors compared with negative priors. For the negative prior, the effect sizes are closer to zero for the same reciprocation rates. In other words, the reciprocation rate has less of an effect on the learning effect size when a negative prior is present compared with a neutral or positive one.

**FIGURE 6:**
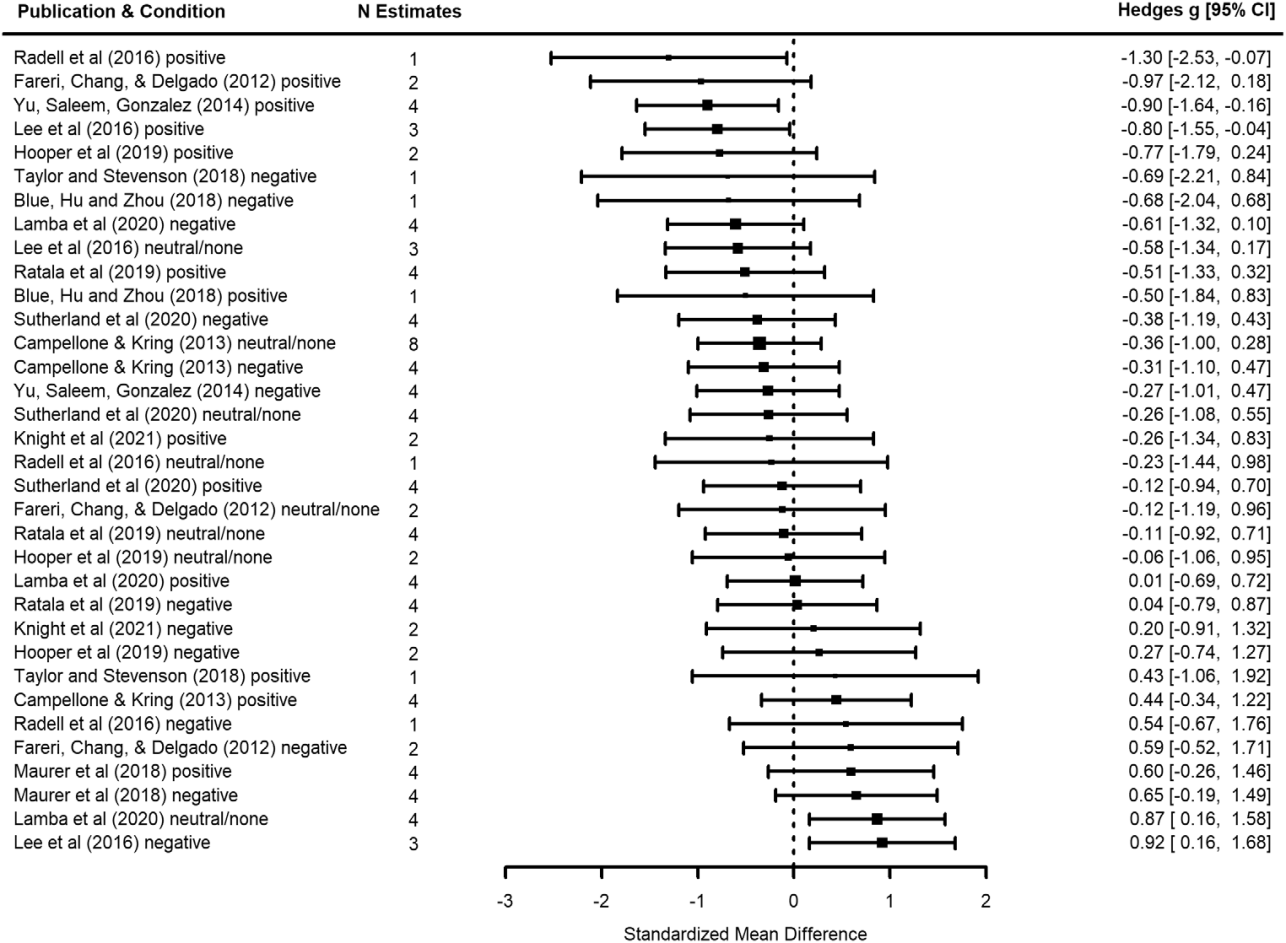
Forest plot of effect sizes pooled by publication and trustworthiness/morality prior for the trustworthiness/morality model. Plotted are the pooled Hedges' *g* and 95% CI for each study, participant group, and condition for the trustworthiness/morality model. *N* estimates = number of effect sizes for a given study and trustworthiness/morality prior, for example, negative, positive or neutral/none. An effect around 0 indicates very little change in trust behaviour or learning occurred. A negative Hedges' *g* indicates participants invested less over time (learning to trust less); a positive Hedges' *g* indicates participants invested more over time (learning to trust more).

To further illustrate the effects, Figure 6 shows the pooled effect of priors within each publication and prior manipulation condition. As can be observed, the values vary widely across and within publications, again pointing to the importance of the reciprocation rate as the most explanatory variable for learning. It also shows that the overall learning rate was slightly negative – meaning people had a tendency, overall, to invest less over time with their partners.

#### Clinical populations

As stated in the preregistration, we only performed analyses for the clinical groups for which we had at least three publications, which happened for psychosis and schizophrenia. Both models showed a positive, statistically significant effect for the reciprocation rate, but no difference between the participant groups based on a clinical diagnosis. See Tables S4 and S5 for detailed results.

#### Priors and reciprocation rate across all studies – exploratory

We conducted an exploratory analysis with all collected studies to assess the effect of negative, positive, or neutral priors about the partners' trustworthiness apart from their behavioural trustworthiness. This is an extension of the previous analyses to examine if there is an effect of positive/negative priors on trust learning, regardless of the specific *type* of those priors. This included all 404 effect sizes from 68 studies.

As observed in Table 2, there is no statistically significant interaction effect of the reciprocation rate and the priors; however, there is a large, positive, statistically significant main effect of the reciprocation rate.

The above model used the neutral/none as the baseline for comparing the priors' effects and how they interact with the reciprocation rate. To extend this, we additionally re‐ran the model with the contrast between positive and negative priors and found no statistically significant interaction effect with the reciprocation rate, but a statistically significant negative main effect for the difference in positive and negative priors (Table S6).

Taken together, there is a main effect of the reciprocation rate and a difference between positive and negative priors, but neither of them differs from the neutral/none prior. Additionally, when removing the interaction term and testing purely for main effects (a common approach when interactions are not significant), these results are further confirmed: negative priors having more positive learning rates than positive or neutral/none priors (Table S7; Figure S3).

This analysis indicates that participants have a more positive learning rate for partners with a negative prior than those with a positive prior, given an equal reciprocation rate. More specifically, with a negative prior at a low reciprocation rate, participants still have a negative learning rate (investing less over time), but it is less negative than with a positive prior, indicating less learning. For a negative prior at a high reciprocation rate, participants have a positive learning rate (investing more over time) and it is more positive than with a partner with a positive prior, indicating more learning. At a meta‐analytic level, it suggests there is less learning when a partner is portrayed negatively and behaves negatively, but more learning when a partner is portrayed negatively but behaves positively.

#### Reciprocation rate definitions – exploratory

**FIGURE 7:**
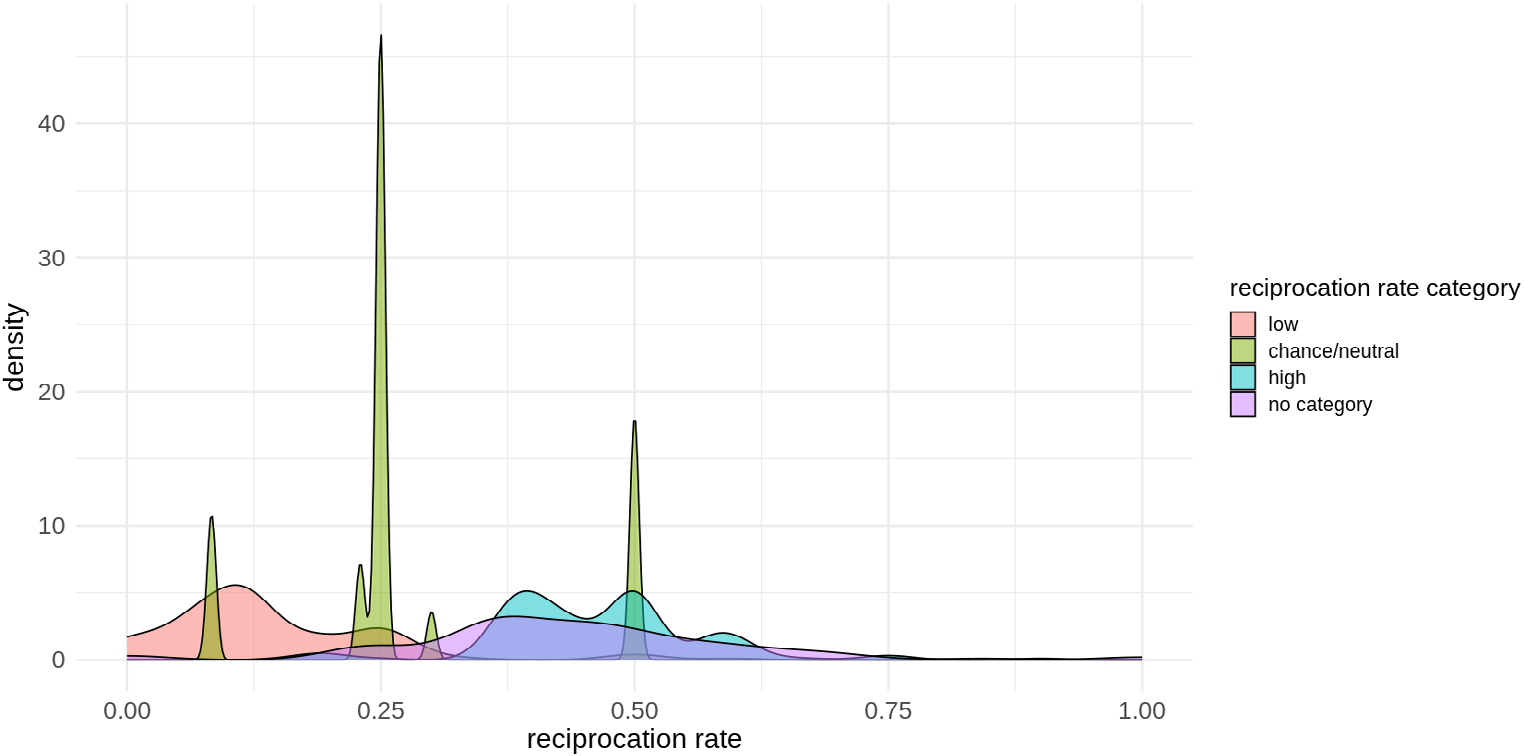
Density plot of the study‐defined reciprocation rate categories. The reciprocation rate or type of partner was defined in many studies, for example, for 307 out of the 404 effect sizes collected, defined in the study as either high/fair (*N* = 149), low/unfair (*N* = 134) or chance/neutral (*N* = 24). The remaining were not categorized (*N* = 97); an example is ‘live’ games in which participants interact freely and reciprocation is therefore not always categorized.

Lastly, we performed an exploratory analysis on how the reciprocation rates, defined as the proportion of the multiplied investment returned, were categorized or labelled in the studies themselves, and how this compares to the distribution we collected. Figure 7 shows the distribution of reciprocation rates categorized by how they were defined in the studies. As can be seen, there is quite some overlap of ‘chance/neutral’ reciprocation rates with both ‘low’ and ‘high’ reciprocation rate categories from other studies. The figure demonstrates that reciprocation rate categories (high/fair, neutral/chance, low/unfair) are very much study‐specific, and these categorizations are thus not comparable across studies.

## DISCUSSION

In the present meta‐analysis, participant characteristics, game design, and their effects on trust learning, as defined by participants' change in trust (investment) behaviour, in the repeated TG were analysed. Our findings indicate that participant trust learning in the repeated TG is driven by their partners' behavioural trustworthiness, as defined by their reciprocation rate, and other factors play a minimal or inconsistent role, conflicting with previous meta‐analytic findings on the single‐shot (one round) TG.

The subgroup analyses examined commonly used TG manipulations to address conflicting findings in the literature about the extent to which participants learn to trust their partners in the repeated TG. The results show that the *type* of manipulation matters in terms of the effects that can be observed, especially in terms of how prior beliefs (induced by experimenter manipulation) interact with the reciprocation rate. Despite some differences, the reciprocation rate was still the most important factor in participants' learning.

### Main model

The full features model which included all pre‐registered and exploratory TG and participant parameters showed that the reciprocation rate was the most important factor in participants' learning, as hypothesized. Additionally, live games were associated with a negative effect (i.e., participants investing less over time) compared with programmed games, reflecting the endgame effect found in tit‐for‐tat strategies in live versions of the repeated TG (Engle‐Warnick & Slonim, 2004). We did not find any statistically significant effects of participant characteristics or other TG features found in a previous meta‐analysis of the single‐shot game (Johnson & Mislin, 2011).

This points to differences in the single‐shot versus repeated TG: in the latter, there is the opportunity for reputation building (Chang et al., 2010; Phan et al., 2010), and due to this learning, factors that influence the single‐shot TG do not influence the repeated TG in the same way. Telga and Lupiáñez (2021) compared behaviour in the repeated TG between young and old participants and found participants' age played a role in the initial tendency to trust (like the single‐shot game) but crucially not in trust learning. Furthermore, it has been shown that when comparing participant behaviour in a single‐shot versus a repeated TG, the amount invested by the trustor is higher in the repeated game than in the single‐shot game (Cochard et al., 2004; Lenton & Mosley, 2011), indicating a more fundamental difference in how participants interact in the single‐shot versus repeated TG.

### Subgroup analyses

#### Group membership manipulation

The group membership analysis showed no effect of group membership on learning. We did not find any evidence for an ingroup preference counter to a substantial amount of literature that shows an ingroup bias is present in the (single‐shot) TG (Balliet et al., 2014; Stanley et al., 2011).

This is, however, not surprising considering the studies with the repeated TG have found conflicting results. For example, Fujino et al. (2020) found participants invested more with the ingroup whereas Vermue et al. (2018) and Telga et al. (2018) found participants invested more with the outgroup. Yet, in both studies, participants still adjusted their investment behaviour towards their partners' trustworthiness (Telga et al., 2018; Vermue et al., 2018). As such, it may be that ingroup bias is observed consistently in the single‐shot TG, but in the repeated TG participants pay most attention to the behavioural evidence of partner trustworthiness and group membership matters less.

#### Social closeness manipulation

The social closeness manipulation analysis examined the effect of social closeness on trust learning and revealed no significant effects, including no effect for the reciprocation rate. This analysis went against our expectation that closeness would have an effect on learning – specifically that one would learn *less* from someone close because of the preferential bias you have toward them (Fareri et al., 2015).

There were far fewer studies than expected that fit the criteria for this subgroup analysis, including just 27 effect sizes (from 9 experiments from 3 studies). As such, the results should be interpreted with caution as they were likely underpowered.

Additionally, the social closeness construct was not standardized across the different studies – for example, only one study included partners that were friends or family (Fareri et al., 2015), whereas ‘close’ in another study (Walasek et al., 2019) meant a fictional partner participants had previously interacted with. It could be that these sublevels of closeness elicit different responses from participants, and therefore, the ‘close’ category in our classification did not capture the hypothesized closeness effect.

#### Trustworthiness/morality manipulation

The analysis of trustworthiness/morality prior manipulations revealed an interaction effect of priors and the reciprocation rate. Specifically, the learning rate tended to be smaller with a negative prior compared with when positive and neutral priors were present. In other words, participants changed their investment behaviour less over time with a negative trustworthiness/morality prior compared with positive and neutral priors.

An explanation for this is a combination of loss aversion and subsequent avoidance. Loss aversion is the phenomenon that humans are more sensitive to potential losses than to gains in risky decision‐making (Kahneman & Tversky, 1972; Sokol‐Hessner & Rutledge, 2019; Tversky & Kahneman, 1992). A negative prior that someone is untrustworthy may mean participants are less likely to invest/trust early on. A negative belief that causes initial avoidance can beget more avoidance and consequently inhibit learning (Allidina & Cunningham, 2021), which could explain the effect of less learning for negative (untrustworthy/immoral) priors. In terms of a Bayesian model, it has been found that people tend to process information faster/perform tasks faster when it fits with stereotypes (less updating needed), but learn more from counter‐stereotypes (more updating needed) (Falbén et al., 2019, 2023), which also explains the observed effect.

#### Priors and reciprocation rate across all studies

We performed an exploratory analysis examining the effect of negative, positive, and neutral/no priors and their interaction with the reciprocation rate across *all* studies, regardless of the type of priors that were present. The model revealed no interaction effect, but a main effect of a more positive learning rate for negative priors compared with positive priors. Thus, participants tended to invest more with partners over time with the negative prior compared with those with the positive prior.

One explanation is the interaction effect coming from the trustworthiness/morality studies which is subsequently weakened by the lack of effect from the other types of manipulations. However, it is important to note that these results do not necessarily conflict: in both analyses, under low reciprocation rates, the negative prior is associated with a more positive learning rate (less change in behaviour) compared with positive priors. In other words, the learning was larger for positive priors compared with negative priors when reciprocation was low in both analyses. Under high reciprocation rates, the analyses show a different effect, with the trustworthiness/morality negative prior having a smaller learning effect compared with positive and neutral priors, and across all studies, the negative prior having a larger learning effect compared with positive priors.

### Summary

In sum, the type of manipulation affects how priors interact (or do not) with the reciprocation rate in shaping how much participants change their trust behaviour in the repeated TG. In particular, the trustworthiness/morality manipulation leads to less learning for negative priors. Across almost all models, however, the reciprocation rate was positively, statistically significantly related to the learning rate, indicating that participants updated their prior beliefs about their partners' behaviour.

The results across models, including the main model, can be explained in terms of a Bayesian framework where the prior beliefs are updated by evidence – the partner's reciprocation. The trustor has a prior belief distribution over the amounts that a trustee will send back (a belief about their trustworthiness), conditional on the amount sent to them (their actual trustworthiness). Given that the trustor is trying to maximize their earnings in the TG (an expected valuation function) over time, the responses of the trustees will result in a Bayesian updating of the trustor's conditional belief distributions. Importantly, we found experimental manipulation of priors to influence trust learning in trustworthiness/morality TG manipulations, but not in social closeness or group membership. One explanation is that trustworthiness/morality manipulations are more effective at changing trust behaviour than other manipulations. Initial (un)trustworthiness manipulations have been shown to have an effect throughout the repeated TG (Chang et al., 2010; Hooper et al., 2019; Yu et al., 2014), indicating the strength of the manipulation. This also points to the trustors' using the initial information about their partners strategically rather than making decisions on preference, which shapes their trust learning. For example, two extensive studies on ingroup favouritism in cooperation games have shown that a core aspect of treating the ingroup preferentially is the expectation that the ingroup will cooperate more with you and not a mere preference for the ingroup's image (Balliet et al., 2014; Romano et al., 2017). In a trustworthiness/morality manipulation, utilizing this information is even more relevant for making strategic decisions maximizing gains.

Another explanation is the data: all but four of the reciprocation rates were at 50% of the participants' multiplied investment *or less*, meaning that most reciprocation rates were low in this analysis. Therefore, participants may not have changed their investment much because the behaviour (low reciprocation) matched the participants' expectations (induced by a negative prior). Taken together, we may observe an effect in the trustworthiness manipulation because (1) the manipulation is effective and (2) reciprocation rates were generally low, meaning partners behaved according to participants' negative expectations for untrustworthy partners, reflecting the finding in Bayesian analysis that people learn less from stereotypes than from counter‐stereotypes (Falbén et al., 2019, 2023).

Although the Bayesian framework for belief updating is useful, human decision makers do not tend to follow the Bayesian equation as‐is (Achtziger et al., 2014). Rather, they may either overweight their existing beliefs – conservatism – or overweight the new evidence, referred to as the representativeness heuristic or base‐rate neglect (Achtziger et al., 2014; Edwards, 1968; Kahneman & Tversky, 1972). Moreover, certain individual characteristics such as the willingness to be incorrect and cognitive capability to recognize and integrate feedback can influence belief updating (Olcaysoy Okten et al., 2023; Rollwage et al., 2018). To assess this more completely, future work using Bayesian modelling on learning in the repeated TG can shed light on why certain manipulations produce different effects.

### Limitations and directions for future study

A methodological consideration is that we examined learning as a standardized mean difference effect. Learning could also be examined in terms of a correlation value which would take into account more data points, that is, investment behaviour correlating with trial number, as opposed to the change in trust on the first and last trials. Both present advantages and disadvantages in terms of how ‘learning’ should be defined; our approach was meant to be more flexible to allow for studies that used pre‐post trust ratings; however, there were far fewer of those than anticipated. The difference between these approaches could be explored in the future.

In terms of experiment design features, there are several areas of interest for future research. Although the number of trials was a statistically insignificant effect in the main model, another useful analysis could be to analyse the extent of learning at different points during the repeated TG, for example, look at the change in trust investment after two trials versus 10 trials and compare this across studies. This could help identify a potential inflection point when participants begin to learn more, given a controlled reciprocation rate. Additionally, we included a variable if participants received a real monetary reward for their performance in the TG as opposed to no TG‐based reward and found no effect, which contradicts the finding by Johnson and Mislin (2011) that random payouts had a negative effect on investment compared with a systematic, TG‐based payout. However, it is important to note our variable is not the exact same and the TG rewards used in the different studies varied considerably, including cumulative earnings (Wang et al., 2021), proportion or ratios of points and their conversion to real money (Goeschl & Jarke, 2017; Maurer et al., 2018), and randomly selected trials, for example, three trials (Alguacil et al., 2017), 10 trials (Blue et al., 2018) and 2 trials (Fareri et al., 2015). A more parsimonious approach to analysing the different types of TG‐based rewards may yield different results, in particular if there is a difference between cumulative earnings versus randomly selected trials. Furthermore, future studies could examine the effect of rewards in more detail, for example, the size of the reward relative to the participants' endowment or purchasing power, or having additional compensation beyond TG performance.

Considering reciprocation rates, the exploration of ‘high, low, medium/chance’ categories as defined by authors in their respective studies is inconsistent at a meta‐analytic level. We suggest that authors designing algorithms for the reciprocation rate not only pay attention to the relative difference between fair and unfair or high and low, but also how the reciprocation rate stacks up against a larger distribution, for example, a ‘high’ reciprocation rate is one in which participants should receive, on average, more than half of what they invested.

Furthermore, the reciprocation rate used in this study was the proportion of the multiplied amount that the partner returned to account for the multiplication factor in considering the trustee's trustworthiness. It has been shown that participants scale their trust offers according to the multiplication factor (Lenton & Mosley, 2011). However, the meta‐analysis by Johnson and Mislin (2011) found that the multiplication factor did not affect trustor behaviour, but resulted in lower trustee reciprocity. Therefore, in this study which focused on participants' learning of trustees' reputation and trustworthiness, we defined the reciprocation rate as the proportion returned of the multiplied investment. However, creating a reciprocation rate definition that is comparable across studies is not trivial. Other definitions of the reciprocation rate, including the return on investment on original investment, which takes a more earnings‐based perspective for the trustor, and how this compares to the definition used in this study, would be important future work.

Lastly, most models showed a statistically significant effect of heterogeneity of the studies. However, the purpose of the meta‐analysis was to identify overarching effects from particular game designs/features, over a broad sample of TG studies, and therefore heterogeneity was anticipated. An avenue for future work is to conduct meta‐analyses that are more homogeneous to drill down further into specific manipulation effects, for example, the type of ingroup‐outgroup.

## CONCLUSION

The meta‐analytic results demonstrate that the most important factor in participants' learning is their partners' reciprocation rate, or in other words, the trustworthiness the partners demonstrate through their behaviour over time. We do not replicate the meta‐analytic effects of TG features and participant characteristics in the Johnson and Mislin (2011) meta‐analytic study of the single‐shot TG, highlighting that the effects on a participant's initial behaviour and their learning behaviour are different. In the repeated TG, trial‐by‐trial learning as a behavioural indicator matters more than game or participant characteristics. The subgroup analyses show that the *type* of prior information and manipulation has different effects on learning. Specifically, there is a lack of an effect for social closeness and group membership on learning in the repeated TG. However, when it comes to trustworthiness/morality manipulations, negative priors are associated with learning less (changing investment behaviour less). When pooling all studies together across all types of manipulations, negative priors compared with positive (but not neutral) were also associated with learning less under low reciprocation, but learning more under high reciprocation.

## AUTHOR CONTRIBUTIONS

**Caitlin Duncan:** Conceptualization; investigation; methodology; formal analysis; writing – review and editing; visualization; validation; writing – original draft; data curation; software. **Lorena Sganzerla:** Data curation; investigation; writing – original draft; writing – review and editing; methodology. **Laura Kaltwasser:** Conceptualization; investigation; writing – review and editing; methodology; validation. **Isabel Dziobek:** Supervision; writing – review and editing; conceptualization; methodology.

## FUNDING INFORMATION

This research received no specific grant from any funding agency in the public, commercial, or not‐for‐profit sectors.

## CONFLICT OF INTEREST STATEMENT

The authors declare no conflict of interest.

## Supporting information


Data S1:

## Data Availability

The data that support the findings of this study are openly available in Trust learning in the repeated Trust Game: a meta‐analytic study at: https://osf.io/nemq3/overview.

## Footnotes

^1^
Forced‐choice tasks are tasks in which a participant is forced to make a choice between alternatives presented, usually 2–4. The task is typically designed in a way that there is a correct choice and participants' accuracy in their ability to discriminate between beneficial and non‐beneficial choice(s) is measured. One example is the Iowa Gambling Task, IGT (Bechara et al., 1994) in which participants try to obtain high rewards by choosing the best of four choice options.

^2^
The Pearson's correlation *r* is necessary for calculating an unbiased Hedges' *g* effect size (Lakens, 2013).

^3^
Studies or experiments that were included in multiple publications were only counted once.

^4^
In three studies, the same participants were used in two different experiments (each of which contained multiple conditions) which we count as unique experiments. Fareri et al. (2012) had the same participants perform the experiments twice separated by a week; Fujino et al. (2020) had two sessions with the same participants: once with TMS and once with TMS sham; Rățală et al. (2019) had two sessions with the same participants: once with participants consuming Ritalin and once with participants consuming a placebo.
