# Supplementary material for: Trust learning in the repeated trust game: A meta‐analytic study
Source: Br J Psychol. 2025 Dec 15;117(3):1026–55. doi: 10.1111/bjop.70045 (PMC13353256; doi:10.1111/bjop.70045)
Supplement: Supplementary file 1 — Data S1: [file BJOP-117-1026-s001.pdf]

Descriptive Results

SI Table 1

Descriptive Statistics of Experiments in Meta-Analysis

Continuous Variables					
Variable	*n*	*M*	*M (SD)*	*Mdn*	*Min - Max*
Reciprocation rate	404[a]	0.34	0.19	0.38	0 - 1
Multiplication factor	167	3.13	0.72	3	1 - 5
Conditions	167	2.40	1.90	2	1 - 8
Trials	167	50.8	91.5	19	2 - 512
Partners	167	3.40	4.0	2	1 - 24
N participants	164[b]	50.5	56.4	32	5 - 382
Participant age	151[c]	26.8	12.0	23	4.4 - 74.5
Participant proportion female	144[c]	0.53	0.23	0.5	0 - 1
Publication year	68[d]	2017	3.23	2018	2004 - 2021

Categorical Variables		
Variable	*n*	*% total*
Participant student population	164[b]	-
Only students	76	46.3%
No students	50	30.5%
Not specified	32	19.5%
Some students	6	3.7%
Participant clinical population	164[b]	-
Non-Clinical	151	92.1%

Variable	*n*	*% total*
Clinical	13	7.9%
Psychosis	5	3.0%
Schizophrenia	3	1.8%
Autism	2	1.2%
BLA Lesion	1	0.6%
Alzheimers	1	0.6%
Behavioral variant frontotemporal dementia	1	0.6%
Counterfactual Reciprocation	167	-
Included	133	79.6%
Not Included	34	20.4%
Reciprocation Rate Type	167	-
Fixed	72	43.9%
Variable	71	43.3%
Adaptive	21	12.8%
Binary vs. Continuous Investment	167	-
Continuous	114	68.3%
Binary	53	31.7%
Trustor Endowment	167	-
Fixed	155	94.6%
Variable	8	4.8%
First Trial Only	1	0.6%
Partner Endowment	167	-
No endowment	128	76.6%
Fixed endowment	37	22.2%

Variable	*n*	*% total*
Variable endowment	2	1.2%
First trial only endowment	0	0%
Live Game vs Algorithm	167	-
Algorithm	131	78.4%
Live	36	21.6%
Infinite vs Finite	167	-
Finite	144	86.2%
Infinite	23	13.8%
Trust Game Reward	167	-
Reward	137	82.0%
No Reward	30	18.0%
Lab vs. Online	167	-
Lab	147	91%
Online - Amazon Turk	12	5%
Online	8	4%
Country	69[e]	-
Africa	1	1.5%
South Africa	1	1.5%
Asia	8	11.6%
China	5	7.2%
Japan	2	3%
Singapore	1	1.5%
Australia	7	10.1%
Europe	27	39.1%
Austria	3	4.4%

Variable	*n*	*% total*
France	3	4.4%
Germany	3	4.4%
Italy	2	3%
Netherlands	9	13%
Norway	2	3%
Poland	3	4.4%
Spain	3	4.4%
UK	5	7.2%
North America	19	27.5%
Canada	2	3%
USA	17	24.7%
South America	1	1.5%
Brazil	1	1.5%

Note. Some values were calculated on the unique study level (country, publication year), some on the unique participant group level, and some on the unique experiment level.

[a]The reciprocation rate is presented on the effect size level as the reciprocation rate was calculated for each condition in each experiment (other TG or participant information, such as the mean age, stays the same across conditions but the reciprocation rate can vary).

[b]In 3 studies (Fareri et al., 2012; Fujino et al., 2020; Ratala et al., 2019), the same participants were used twice. They are only included once in the demographic information, and consequently why there are 167 experiments but 164 unique participant groups.

[c] Total *N* is less than 167 (the number of unique participant groups) due to missing data for that variable; some studies did not report it all information.

[d] Some studies had multiple publication dates because the data was used in multiple publications. In those instances, the first year of publication was taken.

[e] Vermue et al. (2018) used participants in two countries, UK and Netherlands, so it is counted twice.

Meta-Analytic Results

Full Features Model - Egger's Regression Test

In the table below it can be observed that the intercept of the model is statistically significant, indicating possible publication bias.

SI Table 2

Full features model - Egger's regression test for publication bias

Effect	Estimate	*SE*	95% CI		*p*
			LL	*UL*	
Fixed Effects					
Intercept	-1.364	0.511	-2.369	-0.360	0.008
Hedges' g SE	-0.817	0.697	-2.188	0.553	0.242
RR	2.938	0.202	2.542	3.335	0.000
RR adaptive	-0.138	0.312	-0.751	0.475	0.658
RR variable	0.227	0.248	-0.261	0.714	0.361
RR counterfactual	-0.043	0.221	-0.478	0.391	0.844
Investment Continuous v. Binary	-0.027	0.212	-0.443	0.389	0.899
Multiplication Factor	0.251	0.134	-0.012	0.515	0.062
Endowment first trial only	-0.850	0.728	-2.281	0.582	0.244
Endowment variable	-0.409	0.576	-1.541	0.724	0.478
Partner endowment	-0.217	0.235	-0.679	0.244	0.355
Live game	-0.533	0.321	-1.166	0.099	0.098
Infinite game	-0.464	0.361	-1.175	0.246	0.200

Trust game reward	-0.282	0.220	-0.716	0.151	0.201
N trials	-0.001	0.003	-0.006	0.004	0.626
Continent: Africa	0.283	0.696	-1.086	1.652	0.685
Continent: Asia	0.336	0.321	-0.296	0.969	0.296
Continent: Australia	-0.019	0.268	-0.546	0.508	0.944
Continent: Europe	-0.150	0.223	-0.589	0.289	0.503
Continent: South America	1.268	0.776	-0.259	2.795	0.103
Mean Age	-0.007	0.005	-0.018	0.004	0.213
Proportion female participants	0.056	0.230	-0.397	0.509	0.809
students	0.368	0.213	-0.051	0.786	0.085
Clinical population	0.128	0.148	-0.164	0.420	0.390

Random Effects

	Estimate	*n* levels
Publications (level 1)	0.461	58
Experiment/Participant group (level 2)	0.000	143
Effect size condition (level 3)	0.127	359

Note. Model results of the Egger's regression test for publication bias, which is the multivariate, full features meta-analysis model including a term for the standard error of the outcome variable, learning effect size: Three-level estimates of fixed effects and variance components. Forty-five effect sizes were dropped from the analysis due to NaN values in one or more of the

variables. AIC = 720.1, BIC = 823.1. There was significant moderation of the moderator effects, F(df1=23, df2=335) = 10.5, p < .001 and a significant effect of heterogeneity, QE(df=335) = 668.6, p < .001. RR = reciprocation rate; CI = confidence interval; *LL* = lower limit; *UL* = upper limit.

Effect Sizes by Publication

SI Figure 1

Effect sizes by study - pooled across all conditions within study

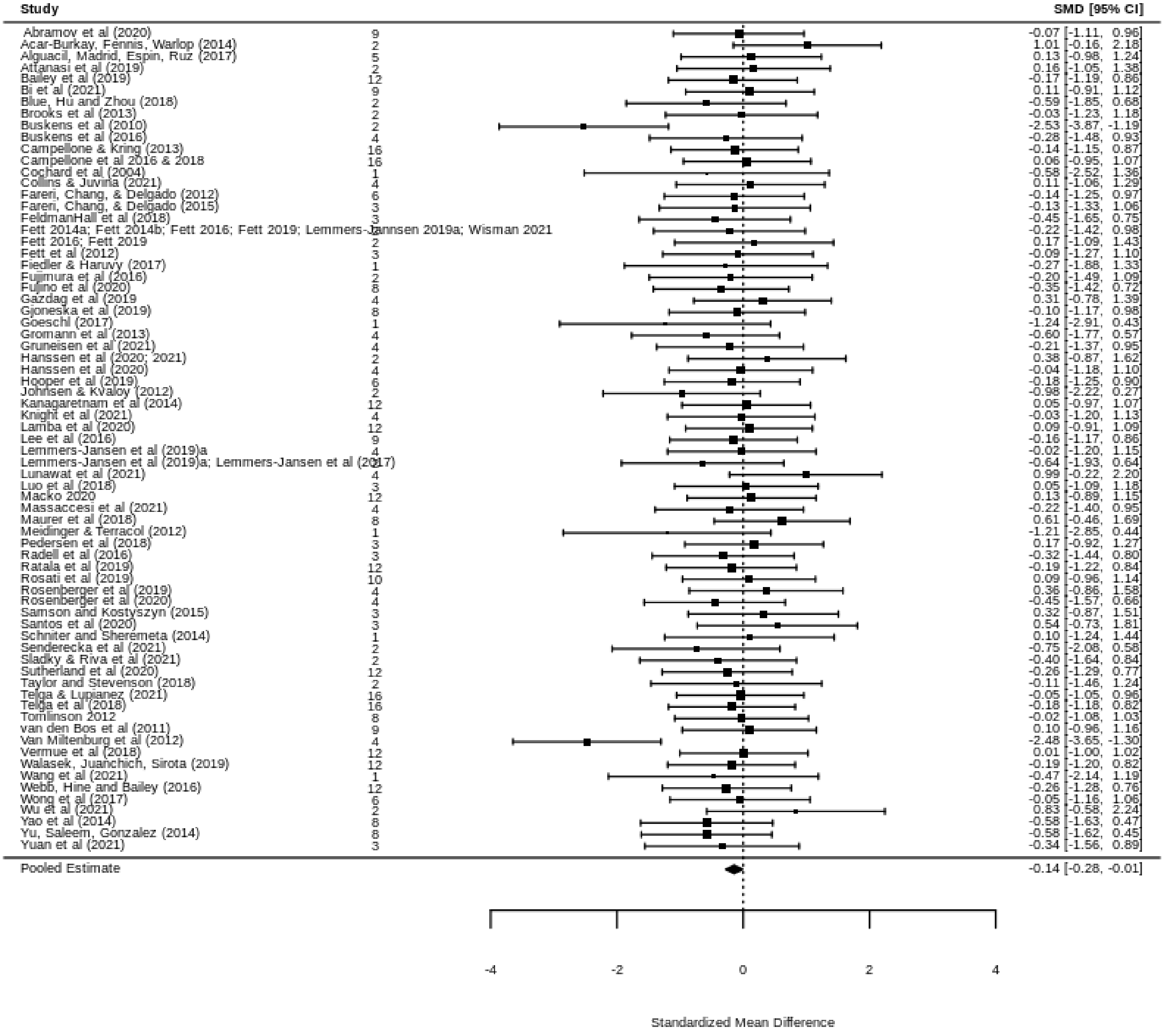

Note. Effect sizes from each publication (or set of publications that used the same data, in some cases). These effects were estimated using a meta-analytic model with three-levels for random effects (publication, participant group, and condition) and a main effect for the reciprocation rate.

The full features model did not include all effect sizes that were collected due to missing values in one of the features included in the model. Therefore, to have an overview of the relative contribution of each publication (or cluster of publications using the same data), we apply a simple model with the reciprocation rate as the main effect and random effects that account for the dependencies in the nested structure of the data.

Social Group

SI Figure 2

Social Group subgroup analysis with interaction term

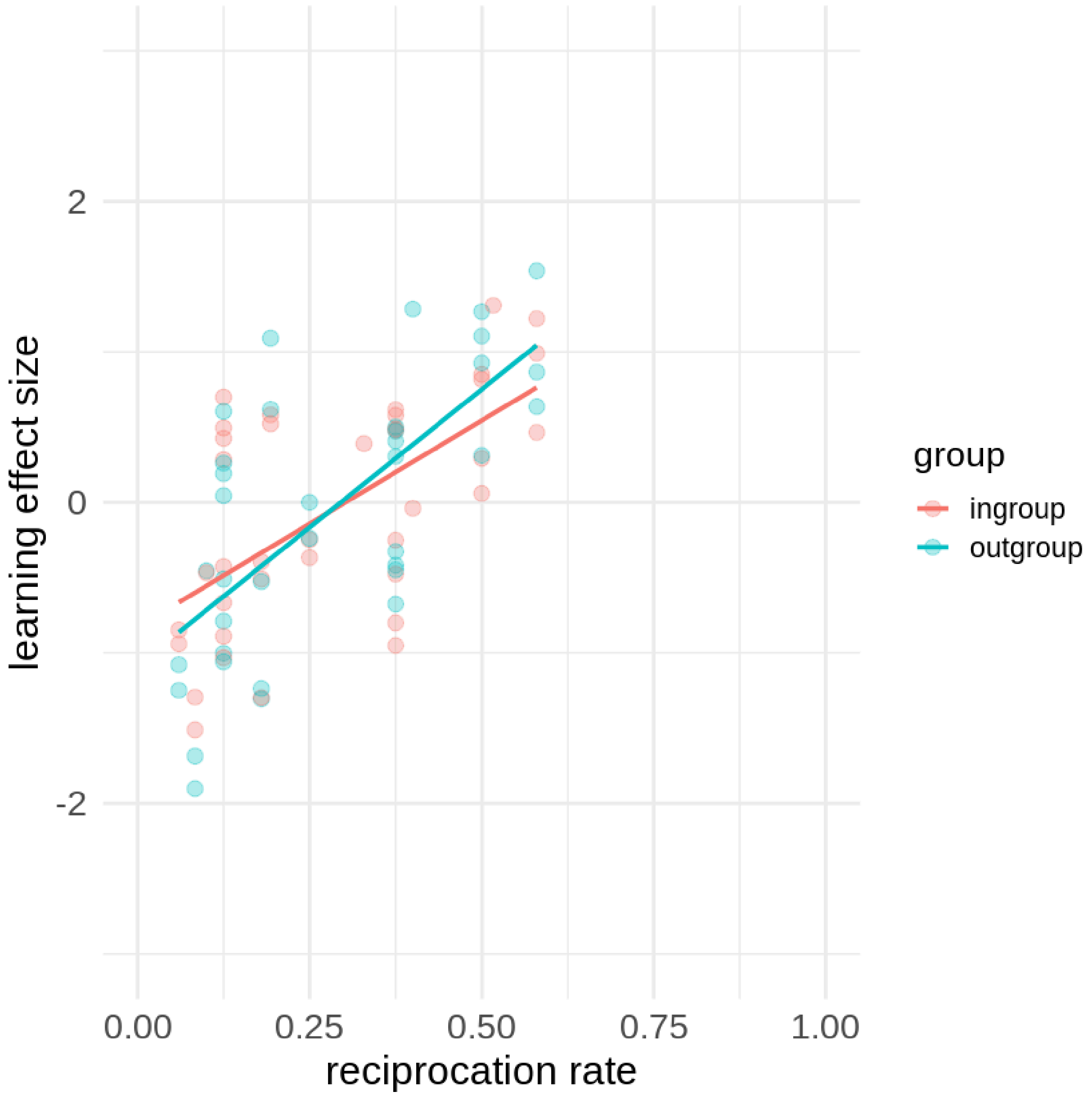

SI Figure 2 displays the linear model of the subgroup analysis including the interaction term. As mentioned in the main text, there is no statistically significant interaction (although it appears to trend in that direction) and no main effect of the partners' group status. There is a main effect of the reciprocation rate (model results in Table 2 in main text).

Social Closeness

SI Table 3

Social Closeness subgroup analysis without interaction effect

Effect	Estimate	*SE*	95% CI		*p*
			LL	*UL*	
Intercept	-0.811	0.270	-1.369	-0.252	0.006
Distant vs. Close	0.147	0.256	-0.382	0.677	0.571
Neutral vs. Close	0.046	0.255	-0.483	0.574	0.860
RR*	1.671	0.634	0.360	2.982	0.015
Random Effects					
	Effects	*n* levels			
Publication (level 1)	0.000	3			
Participant group (level 2)	0.000	9			
Effect size condition (level 3)	0.0.00	27			

Note. AIC = 37.6, BIC = 35.5. There was no statistically significant moderation of the moderator effects, F(df1 = 3, df2 = 23) = 2.73, p = 0.068 and no significant effect of heterogeneity, QE(df = 23) = 10.119, p = 0.991. RR = reciprocation rate; CI = confidence interval; *LL* = lower limit; *UL* = upper limit.

SI Table 3 includes the social closeness model without the interaction term. The reciprocation rate has a statistically significant positive effect. When allowing for an interaction term, there is a trend toward a difference in close vs distant and neutral, rendering a main effect of the reciprocation rate insignificant. However, it could be that there is overfitting with just 5 data points. When excluding the interaction term, which has a better model fit, there is a main effect

of the reciprocation rate. SI Figure 3 demonstrates the effects estimated from the meta-analytic model both with (A) and without (B) an interaction effect in which the clear positive relationship of the reciprocation rate and the learning rate is observed.

SI Figure 3

Linear model with (A) and without (B) the social distance x RR interaction term.

(A) (B)

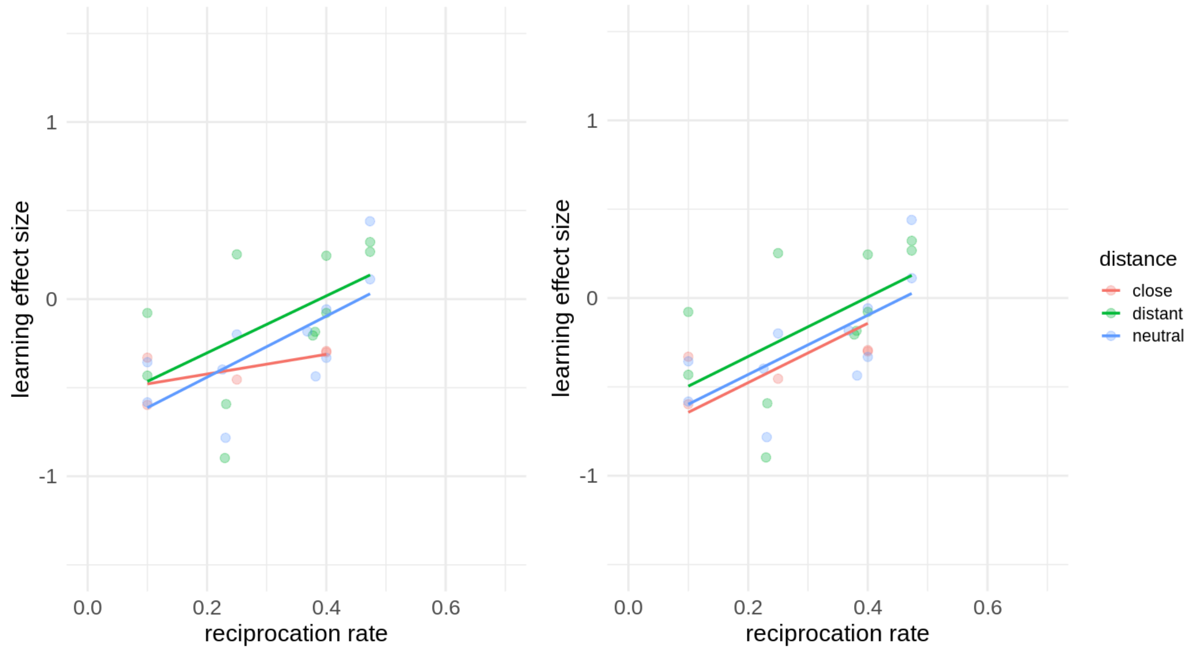

Clinical Groups

As stated in the pre-registration, we only performed analyses for the clinical groups for which we had at least 3 publications. That only occurred for 2 clinical groups, schizophrenia (n=3) and psychosis (n=4). For each subgroup, we tested for an interaction effect of the reciprocation rate and the clinical group vs. nonclinical group. In both analyses, there was no statistically significant difference in learning rates for the clinical groups and the control groups. However, both models showed a statistically significant effect of the reciprocation rate.

Clinical Group: Schizophrenia

The schizophrenia subgroup analysis included data from the following publications: Campellone et al (2016; 2018), Hanssen et al (2020; 2021), Sutherland et al (2020): 3 unique

publications/data sets (Campellone papers had overlapping participants; Hanssen papers had overlapping participants), 7 experiments, 34 effect sizes. The reciprocation rate (RR) had an effect of β = 3.4, SE = 0.81, 95%CI [1.8, 5.1], t(30) = 4.2, p=0.0002. Full results are in SI Table 4.

SI Table 4

Meta-analytic model: participants with schizophrenia vs. neurotypical participants

Effect	Estimate	*SE*	95% CI		*p*
			LL	*UL*	
Intercept	-1.129	0.308	-1.759	-0.500	0.001
RR	3.423	0.809	1.769	5.076	0.000
Schizophrenia vs. Neurotypical	0.427	0.423	-0.438	1.292	0.321
RR x Schizophrenia	-1.742	1.116	-4.021	0.537	0.129
Random Effects					
	Effects	*n* levels			
Publication (level 1)	0.000	3			
Participant group (level 2)	0.000	7			
Effect size condition (level 3)	0.000	34			

Note. AIC = 46.1, BIC = 55.9. There was a statistically significant moderation of the moderator effects, F(df1 = 3, df2 = 30) = 7.974, p =.0005 and no statistically significant effect of heterogeneity, QE(df = 30) = 18.094, p =.960. RR = reciprocation rate; CI = confidence interval; *LL* = lower limit; *UL* = upper limit.

Clinical Group: Psychosis

The psychosis subgroup analysis included data from the following publications: Fett et al (2012), Fett et al (2019), Fett et al (2014a); Fett et al (2014b); Fett et al (2016); Fett et al (2019); Lemmers-Jannsen et al (2019a); Wisman et al (2021), Gromann et al (2013), Lemmers-Jansen et al (2019)a, Lemmers-Jansen et al (2017). Note that many of these publications relied on the TAP study data. In fact, this subgroup analysis included 4 publications without overlapping

participants (in other words, 4 unique publications/data sets), with 10 experiments, and 17 effect sizes. The RR had a positive, statistically significant effect: β = 6.2, SE = 1.5, 95%CI [3.0, 9.4], t(13) = 4.2, p = 0.001.

SI Table 5

Meta-analytic model: participants with psychosis vs. neurotypical participants

Effect	Estimate	*SE*	95% CI		*p*
			LL	*UL*	
Intercept	-2.725	0.602	-4.025	-1.425	0.001
RR	6.238	1.479	3.042	9.433	0.001
Psychosis vs. Neurotypical	0.583	0.827	-1.205	2.370	0.494
RR x Schizophrenia	-0.741	2.087	-5.251	3.769	0.728
Random Effects					
	Effects	*n* levels			
Publication (level 1)	0.029	4			
Participant group (level 2)	0.000	10			
Effect size condition (level 3)	0.000	17			

Note. AIC = 30.4, BIC = 34.3. There was a statistically significant moderation of the moderator effects, F(df1 = 3, df2 = 13) = 10.778, p =.0008 and no statistically significant effect of heterogeneity, QE(df = 13) = 10.104, p =.685. RR = reciprocation rate; CI = confidence interval; *LL* = lower limit; *UL* = upper limit.

Exploratory: Priors x Reciprocation Rate for all effect sizes from all studies

SI Figure 4

Meta-analytic model for priors x reciprocation rate from all effect sizes from all studies

(A) With interaction term *(B) Without interaction term*

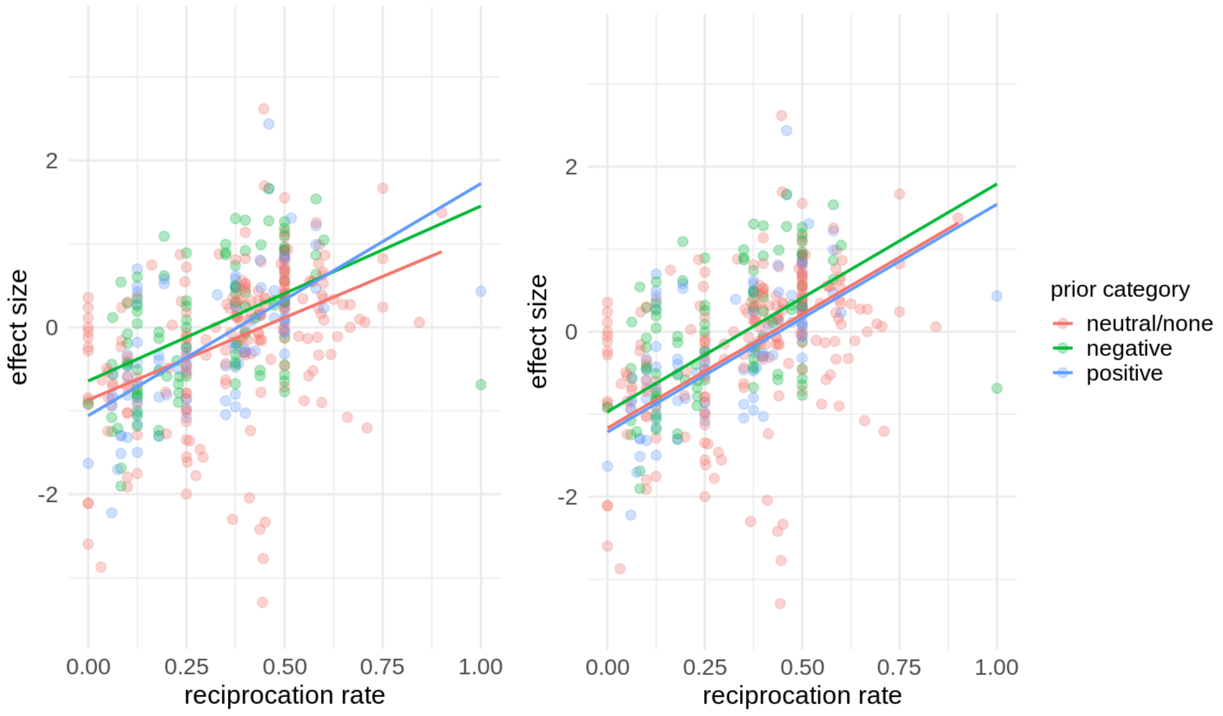

SI Figure 4 shows the meta-analytic model, modeling the relationship between the reciprocation rate and the effect size for each of the prior categories, allowing for an interaction effect (which was not statistically significant). As can be observed, the relationship between reciprocation rate and the learning effect size are parallel for neutral/none and negative priors. The positive prior follows a slightly different trend, however this is not enough for a statistically significant interaction effect. When removing the interaction term from the model, there is a statistically significant main effect of the negative prior having a more positive learning effect than the other two priors; there is no difference between positive and neutral priors in either model.

SI Table 6

Priors and reciprocation rate meta-analytic model for all studies with negative prior as the baseline for comparison.

Effect	Estimate	*SE*	95% CI		*p*
			LL	*UL*	
Intercept	-0.876	0.152	-1.175	-0.578	0.000
Neutral/None vs. Negative	-0.277	0.171	-0.613	0.060	0.107
Positive vs. Negative	-0.475	0.175	-0.820	-0.130	0.007
RR	2.443	0.370	1.715	3.171	0.000
Neutral/None vs. Negative x RR	0.279	0.440	-0.586	1.145	0.526
Positive vs. Negative x RR	0.732	0.490	-0.232	1.696	0.136
Random Effects					
	Effects	*n* levels			
Publication (level 1)	0.244	68			
Participant group (level 2)	0.000	167			
Effect size condition (level 3)	0.126	404			

Note. AIC = 841.2, BIC = 877. There was significant moderation of the moderator effects, F(df1 = 5, df2 = 398) = 43.335, p < .0001 and a significant effect of heterogeneity, QE(df = 398) = 858.482, p < .0001. RR = reciprocation rate, CI = confidence interval; *LL* = lower limit; *UL* = upper limit.

SI Table 7

Priors and reciprocation rate meta-analytic model without interaction effect

Effect	Estimate	*SE*	95% CI		*p*
			LL	*UL*	
Intercept	-1.168	0.105	-1.374	-0.962	0.000
Negative vs. Neutral/None	0.198	0.100	0.002	0.394	0.048
Positive vs. Neutral/None	-0.048	0.098	-0.240	0.145	0.628
RR	2.759	0.191	2.384	3.135	0.000
Random Effects					
	Effects	*n* levels			
Publication (level 1)	0.242	68			
Participant group (level 2)	0.000	167			
Effect size condition (level 3)	0.127	404			

Note. AIC = 841.2, BIC = 877. There was significant moderation of the moderator effects, F(df1 = 3, df2 = 400) = 71.290, $p < .0001$ and a significant effect of heterogeneity, QE(df = 400) = 862.840, $p < .0001$. RR = reciprocation rate; CI = confidence interval; *LL* = lower limit; *UL* = upper limit.
